# Threat intensity shapes cortical engram architecture supporting remote memory retrieval

**DOI:** 10.1038/s41467-026-74231-5

**Published:** 2026-06-11

**Authors:** Miodrag M. Mitrić, Sanne Beerens, Panthea Nemat, Esther Visser, Luca Lorraine van Leeuwen, Rolinka J. van der Loo, August B. Smit, Priyanka Rao-Ruiz, Michel C. van den Oever

**Affiliations:** Department of Molecular and Cellular Neurobiology, Center for Neurogenomics and Cognitive Research, Amsterdam Neuroscience, Vrije Universiteit Amsterdam, Amsterdam, The Netherlands

**Keywords:** Fear conditioning, Spine regulation and structure

## Abstract

The strength of persistent threat memories depends on the intensity of an aversive experience. We combined engram tagging with chemogenetics, electrophysiology and spine analyses in male mice to identify how threat intensity, following mild or strong contextual threat conditioning (CTC), shapes physiological and structural properties of prelimbic cortex (PL) pyramidal neurons. PL engram cells selectively mediate retrieval of a mild remote threat memory, and develop neuroadaptations that are time-dependent, dendritic segment-specific, and modulated by threat intensity. Specifically, increased presynaptic release probability, together with reduced postsynaptic strength and spine size, developed regardless of threat intensity. However, addition of long thin spines occurred exclusively on oblique dendrites of engram neurons after mild CTC, aligning with the PL engram contribution to mild, but not strong, threat memory. Our findings reveal how threat intensity shapes cortical engram architecture, which is pivotal for understanding the neural representation of threat memory strength and persistence.

## Abstract (web summary)

Neural mechanisms underlying remote memory retrieval are not fully understood. This study shows that the intensity of a threatening experience determines whether cortical neurons functionally contribute to remote memory retrieval, in line with threat intensity-dependent synaptic changes on memory-supporting cortical neurons.

## Introduction

Whereas memories of most events fade with time, emotionally charged memories can persist for months-to-years, or even a lifetime. Although threat memory formation is essential for survival, memories of highly aversive experiences can hinder the daily life of affected individuals (e.g., in post-traumatic stress disorder (PTSD)). The adaptive capacity of threat memories is determined by memory strength, with strong threat memories being less adaptive, as exemplified by resilience to extinction of conditioned threat responses^1,2^.

Memory retrieval depends on reactivation of sparsely distributed neurons - engram cells - that were activated during learning^3,4^. These engram cells consolidate physical changes required for memory storage and retrieval^5–7^. As a memory matures over time, memory retention and retrieval become dependent on neocortical brain regions^8,9^. In line with this, engram cells in the PL subregion of the medial prefrontal cortex (mPFC) are necessary for remote ( > 12-day-old), but not recent ( < 1-week-old) threat memory expression^10,11^. This shift is linked to a progressive increase in synaptic strength between PL engram cells after conditioning^12^. Interestingly, we found that the intensity of threat conditioning determines whether PL engram cells are functionally involved in remote memory expression, with strong conditioning resulting in disengagement of the PL engram^10^. In line with this, individuals with PTSD show hypoactivity of the mPFC in response to aversive stimuli, unlike healthy subjects that had been exposed to threat conditioning or patients with social anxiety disorder^13^. The observed disengagement of the mPFC engram after a highly aversive experience may underlie maladaptive memory symptoms, such as resistance to extinction^14^. Hence, understanding the neuroadaptations that develop in PL neurons activated during threat conditioning with different intensities is crucial to identify mechanisms that gate the experience- and time-dependent involvement of the PL engram in remote memory expression, and to elucidate engram signatures underlying differences in memory strength.

Here, we investigated whether temporal changes in electrophysiological excitability and synaptic properties of pyramidal engram cells in PL layer 5 are shaped by the intensity of an aversive experience. In addition, spine density and morphology were compared across distinct dendritic segments of engram and non-engram cells after contextual threat conditioning (CTC). Our data reveal that threat conditioning-activated PL pyramidal neurons (PNs) develop a time-dependent reduction in postsynaptic strength, mirrored by a decrease in spine size, regardless of threat intensity. In addition, spine density on CTC-activated PNs progressively increased exclusively after mild CTC. These results support the differential role of the PL engram in expression of remote threat memories as a function of memory strength.

## Results

### Threat intensity gates engagement of the PL engram in remote memory expression

**Fig. 1:**
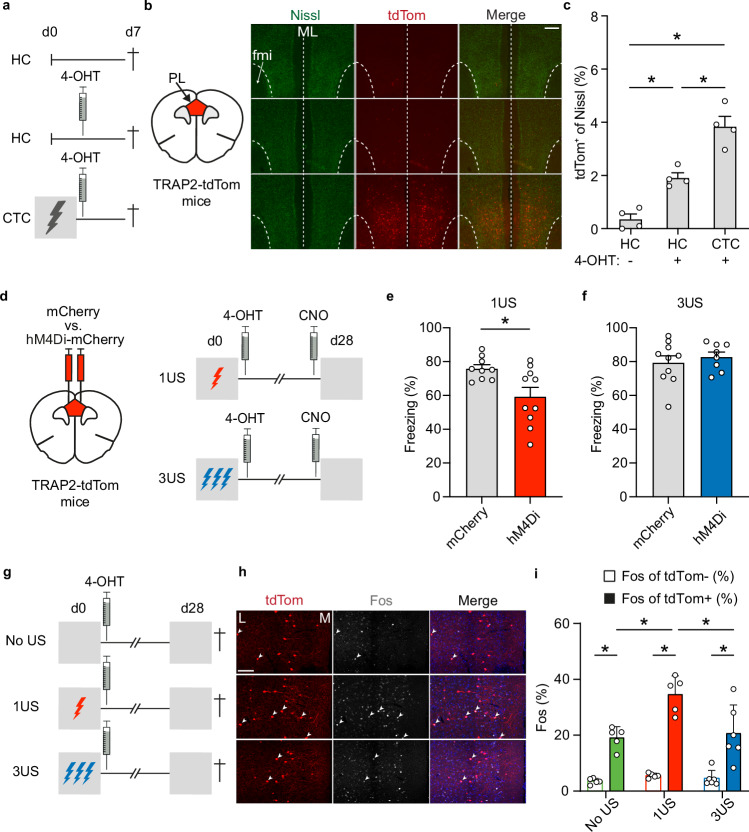
Threat intensity gates engagement of the PL engram in remote memory expression. **a** Experimental design to validate the TRAP2-tdTom mouse line: Home cage – 4-hydroxytamoxifen (HC −4-OHT), HC + 4-OHT, Contextual threat conditioning (CTC) + 4-OHT. Activated neurons were tagged by systemic injection of 4-OHT on day 0 and mice were sacrificed one week later. **b** Expression of tdTom in the PL. fmi = forceps minor of the corpus callosum. Scale bar = 250 µm. **c** Percentage of tdTom^+^ cells in the PL (One-way ANOVA: *F*_2,9_ = 43.7, *p* = 0.000023; post-hoc Bonferroni test: HC −4-OHT *vs*. HC + 4-OHT *p = 0.007, HC −4-OHT *vs*. CTC + 4-OHT *p = 0.000019, HC + 4-OHT *vs*. CTC + 4-OHT *p = 0.002). *n* = 4 mice per group. **d** Experimental design. TRAP2-tdTom mice received AAV-*hSyn*::DIO-hM4Di-mCherry or AAV-*hSyn*::DIO-mCherry in the PL to tag neurons activated during 1US or 3US CTC with hM4Di-mCherry or mCherry alone. CNO was administered 30 min before a remote memory test on day 28. **e** Freezing levels of hM4Di mice were reduced compared with controls after 1US CTC (Unpaired *t*-test: *t*_12_ = 2.8, *p = 0.015). mCherry: *n* = 9, hM4Di: *n* = 10. **f** Freezing levels did not differ between groups after 3US CTC (Unpaired *t*-test: *t*_16_ = 0.6, *p* = 0.56). mCherry: *n* = 10, hM4Di: *n* = 8. **g** Experimental design of the reactivation analysis. No US = no shock, context exposure only. Mice were killed 90 min after the retrieval test. **h** representative images of colocalization of Fos with tdTom^+^ (tdTom^+^/DAPI^+^) and tdTom^-^ (tdTom^-^/DAPI^+^) neurons. **i** Fos preferentially colocalized with tdTom^+^ neurons compared with tdTom^-^ neurons in all groups, but the reactivation level was highest after 1US CTC. (RM ANOVA: Population x Group: F_2,13_ = 7.8, *p* = 0.006; post-hoc Tukey test: No US tdTom^+^ vs. 1US tdTom^+^ *p* = 0.0004, 1US tdTom^+^ vs. 3US tdTom^+^ *p* = 0.0008). Bar graphs show mean + s.e.m. All statistical tests were performed two-sided. For detailed statistical test results, see Supplementary Table 3. Source data are provided as a Source Data file. Illustrations in b and d are adapted from ref. ^10^, used under CC BY 4.0.

TRAP2 transgenic mice were crossed with a tdTomato (tdTom) reporter line to identify CTC-activated PL neurons for electrophysiological recordings and structural analyses. In these mice, activation of the *Fos* promoter drives expression of inducible Cre recombinase (CreER^T2^), which enables 4-hydroxytamoxifen (4-OHT)-controlled recombination of tdTom^15^. We first confirmed that CTC-activated neurons in the PL could be tagged with tdTom in a task-dependent and 4-OHT-controlled manner. TRAP2-tdTom mice remained in their home-cage (HC) in absence or presence of 4-OHT treatment (HC −4-OHT and HC + 4-OHT, respectively) or underwent CTC followed by 4-OHT treatment (CTC + 4-OHT; Fig. 1a). CTC + 4-OHT induced tdTom in 3.9 ± 0.4% (mean ± SEM) of PL neurons (Fig. 1b,c). HC + 4-OHT mice showed less tdTom^+^ cells (1.9 ± 0.2%) and tdTom expression was negligible in HC −4-OHT mice (0.4 ± 0.2%). Next, we investigated in TRAP2-tdTom mice whether CTC-activated PL neurons contribute to remote memory expression after distinct threat intensities. Threat intensity and memory strength can be varied by altering the number of foot-shocks during CTC^2,10^. Here, we trained mice with either 1 or 3 foot-shocks (0.7 mA, unconditioned stimulus (e.g., 1US vs. 3US)), generating a mild or strong threat memory, respectively. In support of this, the 3US threat memory is more resilient to extinction learning than the 1US threat memory (Supplementary Fig. 1). To assess the functional role of 1US and 3US CTC-tagged PL neurons, mice received Cre-dependent AAV expressing hM4Di-mCherry (or mCherry alone) in the PL to enable chemogenetic inhibition of tagged neurons during remote memory retrieval. When mice were re-exposed to the conditioning context on day 28 after training, CNO treatment reduced freezing in hM4Di mice compared with control mice after 1US (Fig. 1e), but not 3US, CTC (Fig. 1f). Remarkably, the 1US group showed high freezing levels that did not differ significantly from the 3US group. We noticed TRAP2-tdTom mice were generally less active than other mouse strains (e.g., C57BL/6 J), which likely contributed to the high freezing levels in both groups. Next, endogenous reactivation of tagged PL neurons during memory retrieval was examined after 1US and 3US CTC, as well as in a No US (no shock control) group (Fig. 1g). Colocalization analysis of Fos (induced during the retrieval test) with tdTom^+^ and tdTom^-^ neurons revealed that tagged PL neurons were preferentially reactivated across all groups, but 1US CTC mice showed the highest reactivation level (Fig. 1h–I, Supplementary Fig. 2a–e). This is in line with the threat intensity-dependent effect of chemogenetic suppression of tagged PL neurons on remote memory expression. Interestingly, the reactivation rate was similar in the No US and 3US CTC groups, suggesting this level may reflect recognition of the context alone. Together, these results confirm that TRAP2-tdTom mice allow tagging of memory-relevant neurons for subsequent electrophysiological and structural analyses, and that CTC-activated PL neurons mediate expression of mild, but not strong, remote threat memory.

### Excitability of tagged PL pyramidal neurons is not altered after mild and strong CTC

**Fig. 2:**
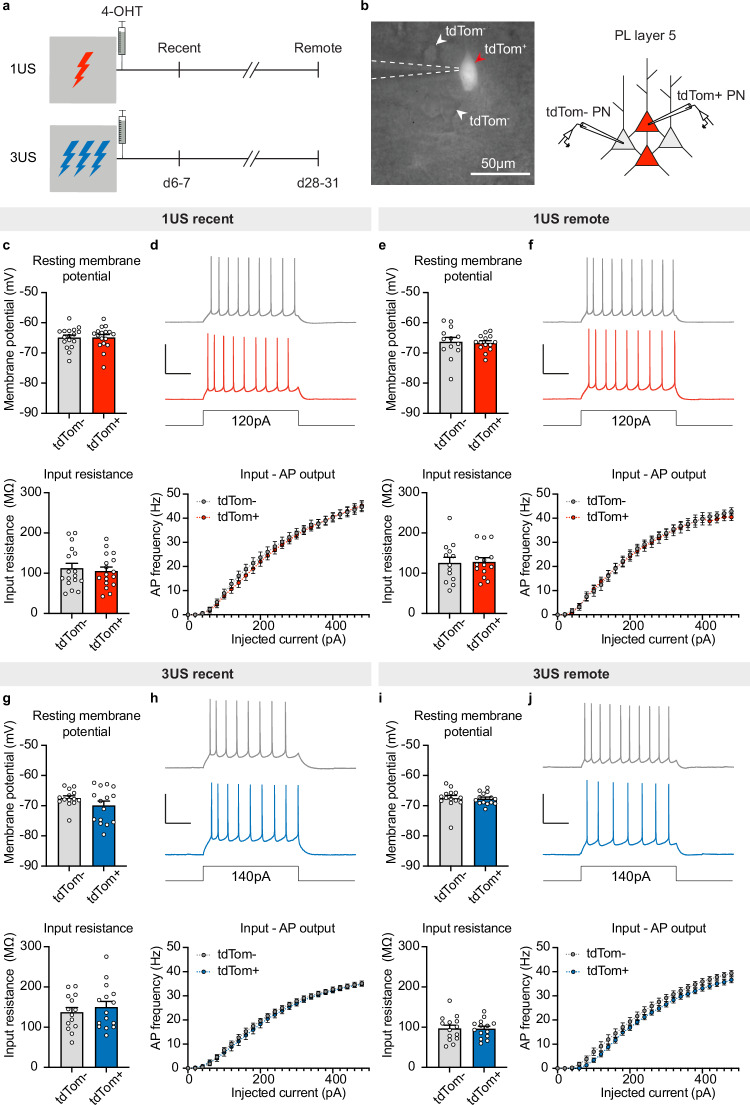
Excitability of tagged PL pyramidal neurons is not altered after mild and strong CTC. **a** Experimental design indicating timepoints when patch-clamp recordings were performed after CTC. **b** Left: image of a patch-clamp recording from a tdTom^+^ pyramidal neuron (PN). Right: schematic of recordings from neighboring tdTom^+^ and tdTom^-^ PNs. **c–d** 1US CTC recent. tdTom^-^ (gray) and tdTom^+^ (red). Both *N*/*n* = 17/4. **c** Resting membrane potential (top; Unpaired *t*-test: *t*_32_ = 0.06, *p* = 0.95) and input resistance (bottom; Unpaired *t*-test: *t*_32_ = 0.4, *p* = 0.66) did not differ between cell populations (**d**) Top: representative trace of action potential (AP) firing in response to 120 pA depolarizing current step in tdTom^-^ and tdTom^+^ neurons. Bottom: AP frequency did not differ between cell populations (Mixed-effects model: Population *F*_1,32_ = 0.2, *p* = 0.66). **e–f** 1US CTC remote. tdTom^-^ N/*n* = 13/4, tdTom^+^ N/*n* = 13/4. **e** Resting membrane potential (top, Unpaired *t*-test: *t*_17.6_ = 0.2, *p* = 0.84) and input resistance (bottom, Unpaired *t*-test: *t*_25_ = 0.2, *p* = 0.88) did not differ between tdTom^-^ and tdTom^+^ neurons. (f) AP frequency did not differ between cell populations (Mixed-effects model: Population *F*_1,25_ = 0.01, *p* = 0.92). **g–h** 3US CTC recent timepoint. tdTom^-^ (gray) N/*n* = 14/3, tdTom^+^ (blue) N/*n* = 15/3. **g** Resting membrane potential (top, Unpaired *t*-test: *t*_20.9_ = 1.5, *p* = 0.16) and input resistance (bottom, Unpaired *t*-test: *t*_27_ = 0.7, *p* = 0.50) did not differ similar between tdTom^-^ and tdTom^+^ neurons. **h** AP frequency did not differ between cell populations (Two-way repeated measures (RM) ANOVA: Population *F*_1,27_ = 0.3, *p* = 0.57). **i–j** 3US CTC remote timepoint. tdTom^-^ N/*n* = 14/4, tdTom^+^ N/*n* = 14/4. **i** Resting membrane potential (top, Mann–Whitney *U* test: *U* = 80.5, *p* = 0.43) and input resistance (bottom, Unpaired *t*-test: *t*_26_ = 0.1, *p* = 0.95) did not differ between cell populations. **j** AP frequency did not differ between cell populations (Two-way RM ANOVA: Population *F*_1,26_ = 2.5, *p* = 0.13. Scale bar: vertical 50 mV, horizontal 200 ms. Bar graphs show mean + s.e.m. Individual data points are neurons. All statistical tests were perfomed two-sided. For detailed statistical test results, see Supplementary Table 3. Source data are provided as a Source Data file.

We assessed whether excitability of CTC-tagged PL neurons changes in an experience-dependent manner at a recent and remote timepoint after learning. PL neurons activated during 1US or 3US CTC were tagged and acute brain slices containing the PL were prepared at a recent (6–7 days) or remote (28–31 days) timepoint after training (Fig. 2a). Mice did not undergo a memory retrieval test to assess learning-induced changes. Whole-cell electrophysiological recordings were performed from layer 5 pyramidal neurons (PNs) given their prevalent cortical and subcortical connections^16^, specific relevance in remote memory expression^17^, and because they have an extensive apical dendrite relevant for structural analyses of distinct dendritic segments. Moreover, of all PL engram cells, the largest fraction is located in the deep cortical layers and reactivated during remote memory retrieval (Supplementary Fig. 2f–g)^15^. The excitability of tagged (tdTom^+^) PNs and neighboring non-tagged (tdTom^-^) PNs was compared (Fig. 2b). Following 1US CTC, the resting membrane potential, input resistance, action potential (AP) discharge frequency, or other measured parameters (Supplementary Table 1) were not different between the two PN populations at the recent timepoint (Fig. 2c, d) and remote timepoint (Fig. 2e, f). Similarly, no excitability differences were observed after 3US CTC at the recent and remote timepoint (Fig. 2g–j, Supplementary Table 2). Although tdTom^+^ neurons showed a small reduction in AP discharge frequency compared to tdTom^-^ neurons (Fig. 2j), this did not reach significance. Thus, mild and strong threat conditioning do not affect the excitability properties of layer 5 PL PNs.

### Reduced postsynaptic strength and enhanced presynaptic release probability in CTC-activated PL PNs

**Fig. 3:**
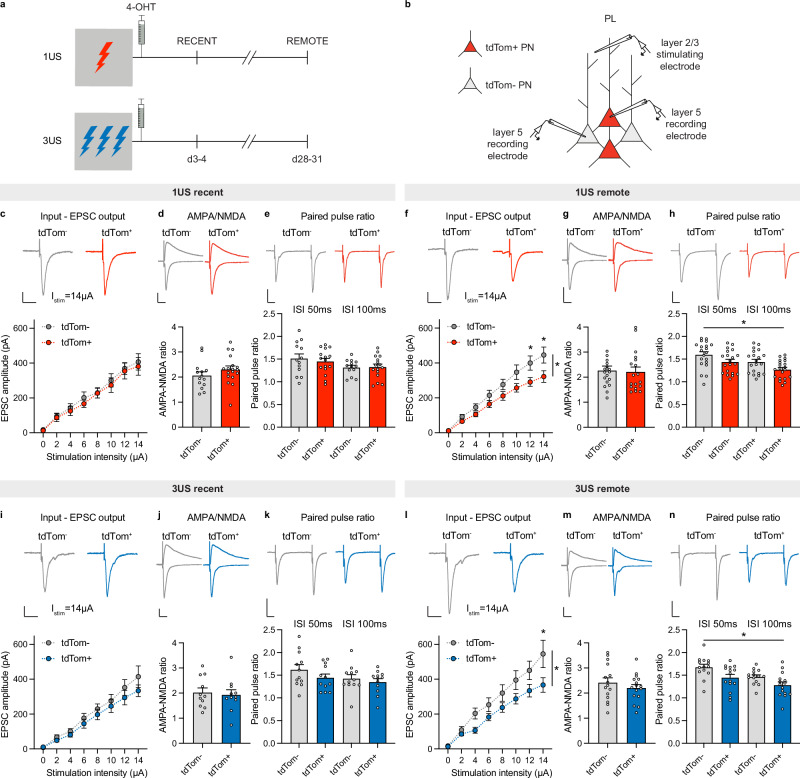
Reduced postsynaptic strength and enhanced presynaptic release probability in CTC-activated PL PNs. **a** Experimental design. Recordings were performed at a recent or remote timepoint after CTC. **b** Illustration of electrode placements. A stimulating electrode in layer 2/3 evoked excitatory postsynaptic currents (eEPSC). **c** 1US CTC recent timepoint. Top: representative traces of eEPSCs in tdTom^-^ and tdTom^+^ PNs. Bottom: eEPSC input-output curve did not differ between cell populations (Mixed-effects model: Population *F*_1,31_ = 0.3, *p* = 0.62). **d** Top: representative eEPSCs recorded at -70 mV (bottom trace) and 40 mV (top trace) to determine AMPAR and NMDAR current amplitudes, respectively. Bottom: AMPAR/NMDAR current ratios did not differ (Unpaired *t*-test: *t*_30_ = 1.2, *p* = 0.23). **e** Top: representative eEPSCs with a 100 ms interstimulus interval (ISI). Bottom: Paired pulse ratio (PPR) did not differ between cell populations (RM ANOVA: *F*_1,27_ = 0.1, *p* = 0.77). **f** 1US CTC remote timepoint. eEPSC input-output curve. Mixed-effects model revealed a cell population effect (Population *F*_1,4_ = 5.3, *p = 0.027; Post-hoc Bonferroni tests: 12 µA *p = 0.028; 14 µA *p = 0.007). **g** AMPAR/NMDAR current ratios did not differ (Unpaired *t*-test: *t*_34_ = 0.2, *p* = 0.85). **h** PPR differed between cell populations (RM ANOVA: Population *F*_1,38_ = 5.1, *p = 0.031). **i** 3US CTC recent timepoint. eEPSC amplitudes did not differ between populations (Mixed-effects model: Population *F*_1,27_ = 1.0, *p* = 0.33). **j** AMPAR/NMDAR current ratios did not differ (Unpaired *t*-test: *t*_20_ = 0.3, *p* = 0.74). **k** PPRs did not differ between populations (RM ANOVA: Population *F*_1,22_ = 1.3, *p* = 0.27). **l** 3US CTC remote timepoint. eEPSC input-output curve. Mixed-effects model revealed a Population x Stimulation interaction (*F*_7,241_ = 2.5, *p = 0.016; Post-hoc Bonferroni test: 14 µA *p = 0.006). **m** AMPAR/NMDAR current ratios did not differ (Unpaired *t*-test: *t*_29_ = 0.9, *p* = 0.37). **n** PPR differed between populations (RM ANOVA: *F*_1,27_ = 6.7, *p = 0.016). Scale: vertical 100 pA, horizontal 20 ms. Bar graphs show mean + s.e.m. Individual data points are neurons. All statistical tests were performed two-sided. For detailed statistical test results, see Supplementary Table 3. Source data are provided as a Source Data file.

We next investigated electrophysiological properties of synapses on CTC-tagged (tdTom^+^) PNs in the PL and compared them to neighboring non-tagged (tdTom^-^) PNs at a recent and remote timepoint after CTC (Fig. 3a). Evoked excitatory postsynaptic currents (eEPSC) were recorded in layer 5 PNs using a stimulating electrode in layer 2/3 (Fig. 3b–c), which was based on axons from neurons in threat memory relevant regions (e.g., intracortical, basolateral amygdala (BLA), thalamic nuclei) that innervate these layers^12,18–20^. We assessed three parameters: (1) eEPSC input–output curves. (2), AMPAR /NMDAR current ratios, and (3) paired pulse ratios (PPR), as an index of presynaptic release probability^21,22^. After 1US CTC, none of the aforementioned parameters differed between the tdTom^-^ and tdTom^+^ PNs (Fig. 3c–e) at the recent timepoint. However, at the remote timepoint, eEPSCs amplitudes were reduced in tdTom^+^ neurons compared with tdTom^-^ neurons (Fig. 3f), without a change in AMPAR/NMDAR ratio (Fig. 3g). Furthermore, the PPR was lower in tdTom^+^ PNs compared with tdTom^-^ PNs (Fig. 3h), pointing to increased presynaptic release probability.

Similar changes were observed after 3US CTC. Synaptic parameters were unchanged between tdTom^-^ and tdTom^+^ PNs at the recent timepoint (Fig. 3i–k). At the remote timepoint, tdTom^+^ neurons showed lower eEPSC amplitudes (Fig. 3m), despite a comparable AMPAR/NMDAR ratio in both populations (Fig. 3m), and tdTom^+^ neurons exhibited a reduced PPR compared to tdTom^-^ PNs (Fig. 3n). To determine whether the synaptic changes observed at the remote timepoint depend on an aversive stimulus during conditioning, we exposed mice to the same context in absence of a foot-shock (No US). Four weeks later, tdTom^+^ and tdTom^-^ PNs showed no difference in eEPSC amplitude, AMPAR/NMDAR ratio and PPR (Supplementary Fig. 3). Hence, all observed synaptic changes developed over time and exclusively after threat conditioning. The eEPSC amplitude was reduced after both mild and strong CTC. As the peak eEPSC amplitude in the input-output measurements (at −70 mV) predominantly reflect the contribution of AMPAR currents, and AMPAR/NMDAR current ratios did not change, both AMPAR and NMDAR currents are likely reduced to a similar extent in CTC-tagged PL neurons, reflecting reduced postsynaptic strength. In contrast, the decreased PPR after mild and strong CTC points to an increase in presynaptic release probability onto PL pyramidal engram cells.

### Spine density progressively increases on oblique dendrites of PL PNs activated during mild CTC

**Fig. 4:**
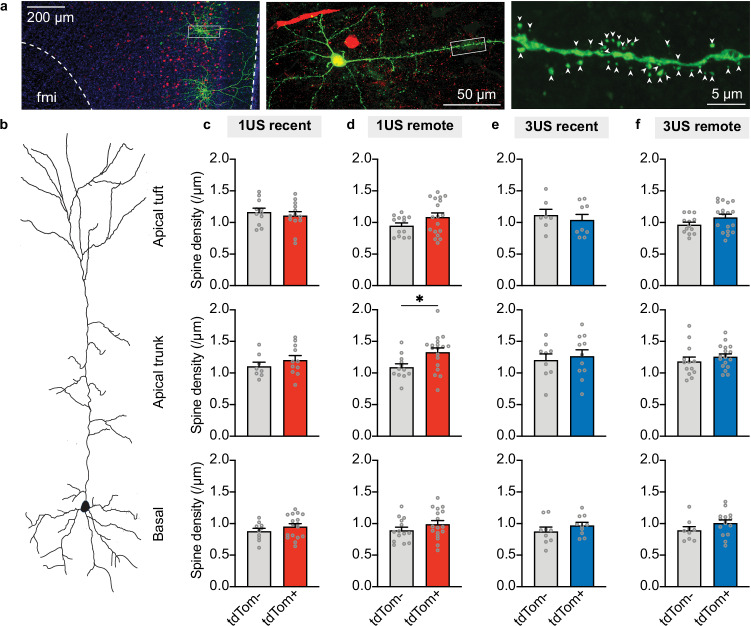
Spine density progressively increases on oblique dendrites of PL PNs activated during mild CTC. **a** Left: example of neurons filled with biocytin (green) during the eEPSC recording. Middle: cell identity was confirmed through colocalization of biocytin with tdTomato (tdTom^+^; yellow in example) or absence of colocalization (tdTom^-^). Right: arrow heads indicate dendritic spines. **b** Schematic of a layer 5 pyramidal neuron with apical tuft, apical trunk and basal dendrites. **c** 1US CTC recent timepoint. Spine density did not differ between tdTom^+^ and tdTom^-^ neurons at all dendritic segments (Mixed effects model: Population *F*_1,29_ = 0.6, *p* = 0.45). **d** 1US CTC remote timepoint. Mixed-effects model revealed a Population effect (*F*_1,30_ = 6.3, *p* = 0.017). Post-hoc Bonferroni test confirmed a difference in spine density between tdTom^-^ and tdTom^+^ neurons at the apical trunk (*p = 0.031), but not at the apical tuft (*p* = 0.27) nor basal (*p* = 0.59), dendrites. **e** 3US CTC recent timepoint. Spine density did not differ between tdTom^+^ and tdTom^-^ neurons at all dendritic segments (Mixed-effects model: Population *F*_1,29_ = 0.1, *p* = 0.75). **f** 3US CTC remote timepoint. Mixed-effects model revealed a Population effect (*F*_1,29_ = 4.7, *p* = 0.039), but post-hoc Bonferroni test did not detect a difference between tdTom^-^ and tdTom^+^ neurons at the apical tuft (*p* = 0.25), trunk (*p* = 1*)* and basal (*p* = 0.47) dendrites. Bar graphs show mean + s.e.m. Individual data points are neurons. For detailed statistical test results, see Supplementary Table 3. Source data are provided as a Source Data file.

Dendritic spines are key postsynaptic structures of excitatory neurotransmission and changes in spine number reflect structural plasticity^23^. To investigate whether CTC-tagged PL neurons exhibit alterations in density of dendritic spines, we filled tdTom⁺ (tagged) and tdTom⁻ (non-tagged) neurons used for eEPSC recordings with biocytin to enable imaging of proximal and distal dendritic segments for spine density analysis (Fig. 4a). As distinct long-range inputs to the mPFC target specific cortical layers^24,25^, we quantified spines across 3 segments of the dendritic arbor: apical tuft dendrites, oblique dendrites on the apical trunk, and basal dendrites (Fig. 4b). At the recent timepoint after 1US CTC, spine density was similar between tdTom^+^ and tdTom^-^ neurons across all dendritic segments (Fig. 4c). In contrast, at the remote timepoint after 1US CTC, tdTom^+^ neurons showed a significant increase in spine density specifically on oblique dendrites at the apical trunk of tdTom^+^ neurons, whereas on apical tuft and basal dendrites spine density remained unchanged (Fig. 4d). Following 3US CTC, no differences in spine density were observed between tdTom^-^ and tdTom^+^ neurons at either timepoint (Fig. 4e, f). The mixed-effects model analysis detected a main effect of cell population at the remote timepoint after 3US CTC, however, post-hoc testing did not reveal a difference in spine density at any of the dendritic segments. Moreover, in mice that did not receive a foot-shock during context exposure, spine density was also unaffected at the remote timepoint (Supplementary Fig. 4). These findings reveal that compared with non-tagged PNs, structural plasticity through increased spine density develops progressively and exclusively on oblique dendrites of tagged PNs after mild CTC, corresponding with their selective functional involvement in retrieval of the mild remote threat memory.

### The frequency of miniature EPSCs is enhanced in PL PNs activated during mild CTC

**Fig. 5:**
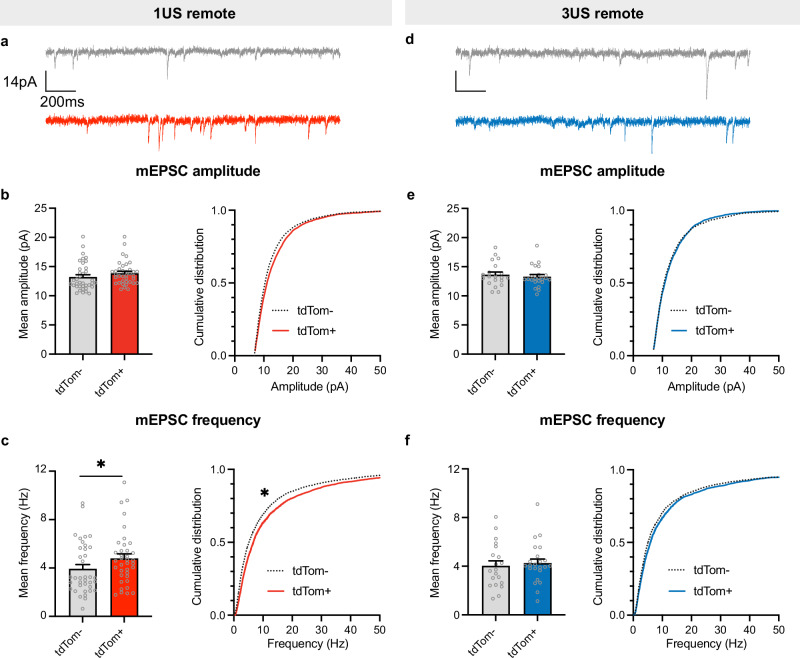
The frequency of miniature EPSCs is enhanced in PL neurons activated during mild CTC. PL neurons activated during 1US CTC (**a**–**e**) or 3US CTC (**f**–**j**) were tagged and miniature EPSC (mEPSC) recordings were performed from neighboring tdTom^+^ and tdTom^-^ PNs in layer 5 at a remote timepoint after CTC. **a** Representative traces of mEPSC recordings in tdTom^-^ (gray) and tdTom^+^ (red) neurons after 1US CTC. tdTom^-^ *N/n* = 37/15, tdTom^+^ *N/n* = 37/15. **b** Left: the mean mEPSC amplitude did not differ between populations (two-sided Mann–Whitney U test: *U* = 510, *p* = 0.06). Right: cumulative distribution plot of mEPSC amplitudes. **c** Left: the mean mEPSC frequency was enhanced in tdTom^+^ compared with tdTom^-^ neurons (one-sided Mann–Whitney U test: *U* = 522, *p = 0.039). Right: the cumulative distribution plot of mEPSC frequencies revealed a rightward shift in the tdTom^+^ population (Kolmogorov Smirnov test: *D* = 0.06, *p* < 0.000001). **d** Representative traces of mEPSC recordings in tdTom^-^ (gray) and tdTom^+^ (blue) neurons after 3US CTC. tdTom^-^ *N/n* = 20^/^8, tdTom^+^ *N/n* = 23^/^8. **e** Left: th**e** mean mEPSC amplitude did not differ between cell populations (two-sided Mann-Whitney U test: *U* = 199, *p* = 0.46). Right: cumulative distribution plot of mEPSC amplitudes. **f** Left: the mean mEPSC frequency did not differ between populations after 3US CTC (two-sided Mann–Whitney U test: *U* = 204, *p* = 0.54). Right: cumulative distribution plot of mEPSC frequencies. Bar graphs show mean + s.e.m. Individual data points are neurons. Color coding of representative traces matches the bar graphs. For detailed statistical test results, see Supplementary Table 3. Source data are provided as a Source Data file.

A change in dendritic spine density typically correlates with a change in frequency of spontaneous excitatory synaptic transmission^26,27^. To investigate whether the increase in spine density on tagged PL PNs after 1US CTC translates to enhanced spontaneous excitatory input in these cells, we recorded miniature EPSCs (mEPSCs) in tdTom^+^ and tdTom^-^ layer 5 PNs at a remote timepoint (28–31 days) after 1US or 3US CTC. First, the means of mEPSC amplitudes or frequencies were compared and only when this reached significance, cumulative distribution plots were compared to demonstrate a potential shift in the curves. After 1US CTC, the mean mEPSC amplitude did not differ significantly in tdTom^+^ and tdTom^-^ neurons (Fig. 5a–c), however, the mean mEPSC frequency was enhanced in tdTom^+^ neurons (Fig. 5d) and accompanied by a rightward shift in the distribution of sampled instantaneous mEPSC frequencies (Fig. 5e). These findings are consistent with the increased spine density observed selectively on oblique dendrites of PL engram cells. In contrast, after 3US CTC, the mean mEPSC amplitude and frequency did not differ between both PN populations (Fig. 5e, g) and their cumulative distribution was similar. Moreover, in mice that were exposed to the conditioning context alone (No US), the mean amplitude and frequency of mEPSCs also did not differ between tdTom^-^ and tdTom^+^ neurons (Supplementary Fig. 5). Thus, both mEPSC frequency and spine density increased on tdTom^+^ PNs exclusively after 1US CTC, suggesting that excitatory synaptic connectivity of PL engram cells is enhanced at a remote timepoint after a mild aversive experience.

### PL engram cells have more long thin spines than non-engram cells after mild CTC

**Fig. 6:**
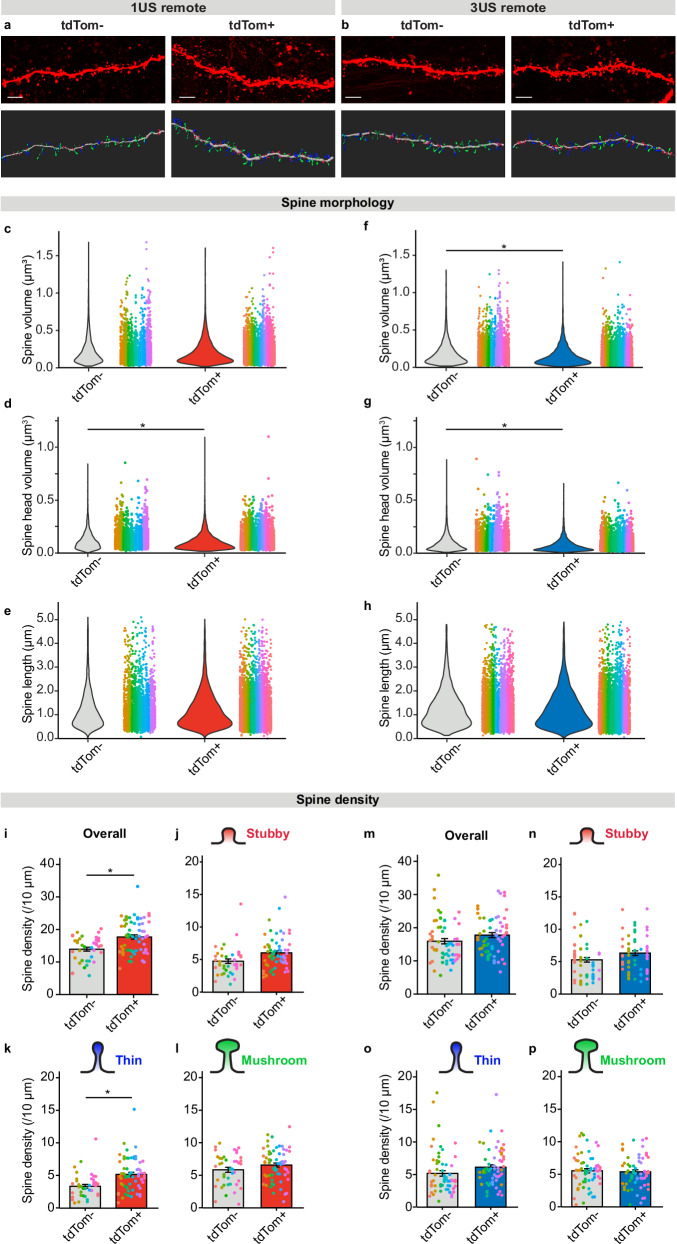
PL engram cells have more long thin spines than non-engram cells after mild CTC. Examples of reconstructions of tdTom^-^ and tdTom^+^ oblique dendrites after 1US (**a**) and 3US (**b**) CTC showing stubby (red), long thin (blue) and mushroom (green) spines. Scale bar = 3 µm. **c–p** Morphology parameters and classification of tdTom^+^ and tdTom^-^ spines after 1US (**c**–**e**, **i**–**l**) and 3US (**f**–**h**, **m**–**p**) CTC were analysed by multilevel regression analysis. Violin plot dots represent spines and colors reflect dendritic fragments (**c**–**h**). Bar graphs show mean +/− s.e.m, dots represent dendritic fragments and colors reflect sections (**i**), cells (**j**–**m**, **o**–**p**) or mice (**n**). **c** Spine volume was unaffected (*χ*^2^(1) = 1.28, *p* = 0.26). **d** Spine head volume was reduced on tdTom^+^ dendrites (*b* = −0.14, −2.05, *p = 0.040). **e** Spine length was unaffected (*χ*^2^(1) = 0.10, *p* = 0.75). **f** Spine volume was reduced on tdTom^+^ dendrites (*b* = −0.12, *t* = −2.13, *p = 0.033). **g** Spine head volume was reduced on tdTom^+^ dendrites (*b* = −0.19, *t* = −2.78, *p* = 0.006). **h** Spine length was unaffected (*χ*^2^(1) = 0.59, *p* = 0.44). **i** Spine density was enhanced on tdTom^+^ dendrites (*b* = 0.25, *t* = 4.14, *p < 0.001. **j** Stubby spines showed a trend towards increased density on tdTom^+^ dendrites (*χ*^2^(1) = 3.50*, p* = 0.062, Predictor model: *b* = 0.26, *t* = 1.94, *p* = 0.053). **k** Long thin spine density was increased on tdTom^+^ dendrites (*b* = 0.46, *t* = 2.66, *p = 0.008). **l** Mushroom spine density was unaltered (*χ*^2^(1) = 0.00*, p* = 1. **m** S*p*ine density did not differ (*χ*^2^(1) = 0.9^2^*, p* = 0.34). **n** Stubby spines showed a trend towards increased density on tdTom^+^ dendrites (*χ*^2^(1) = 3.24*, p* = 0.07, *b* = 0.15, *t* = 1.81, *p* = 0.07). **o** Long thin spine density was unaltered (*χ*^2^(1) = 2.07*, p* = 0.15). ***p*** Mushroom spine density was unaltered (*χ*^2^(1) = 0.00*, p* = *1*). For detailed statistical test results, see Supplementary Table 3. Source data are provided as a Source Data file.

As synaptic activity can drive changes in spine shape and size, and thereby alter synaptic strength and connectivity, we next investigated whether the increased spine density on oblique dendrites of PL PNs activated during mild CTC reflects a change in spine morphology and addition of a specific spine type. For this, we generated 3D reconstructions of oblique dendrites on the apical trunk of biocytin-filled PNs used for mEPSC recordings at a remote timepoint after CTC (Fig. 6a). Spine morphology parameters were analysed using a multilevel regression analysis while controlling for a nested data structure^28^. We first examined general parameters, including total spine volume, spine head volume and spine length. Following 1US CTC, spine volume and length did not differ between tdTom^-^ and tdTom^+^ dendrites, but tdTom^+^ dendrites showed a significant decrease in spine head volume (Fig. 6c–e). After 3US CTC, both spine volume and spine head volume were reduced on tdTom^+^ dendrites, whereas spine length was unaffected in comparison to tdTom^-^ dendrites (Fig. 6f–h). Next, we compared spine density between tagged and non-tagged PNs after 1US (Fig. 6i–l) or 3US (Fig. 6m–p) CTC, and in particular classified the density of specific spine types (stubby, long thin and mushroom) based on their length and the ratio between the volume of the spine head and spine neck^29^. When pooling all spine types, we found that spine density was enhanced on tdTom^+^ compared with tdTom^-^ dendrites after 1US CTC (Fig. 6i, m), confirming the findings of oblique dendrites from neurons in the eEPSC recordings (Fig. 4d). Interestingly, after 1US CTC, stubby spines showed a trend towards increased density and long thin spine density was enhanced on tdTom^+^ compared with tdTom^-^ dendrites (Fig. 6j, k). The density of mushroom spines did not differ between PN populations (Fig. 6l). After 3 US CTC, overall spine density did not differ between PN populations. Similar to 1US CTC, stubby spines showed a similar trend towards increased density on tdTom^+^ compared with tdTom^-^ PNs after 3US CTC (Fig. 6n), likely contributing to the overall reduction in spine volume. However, 3US CTC did not alter the density of long thin spines (Fig. 6o) and mushroom spine density was also unaffected (Fig. 6p). Taken together, the morphological spine signature of PL pyramidal engram cells after mild CTC involved addition of long thin spines. Additionally, the average spine size (total volume and/or spine head volume) on PL PNs that were activated during conditioning was reduced compared with PNs that were not activated, independent of threat intensity.

## Discussion

**Fig. 7:**
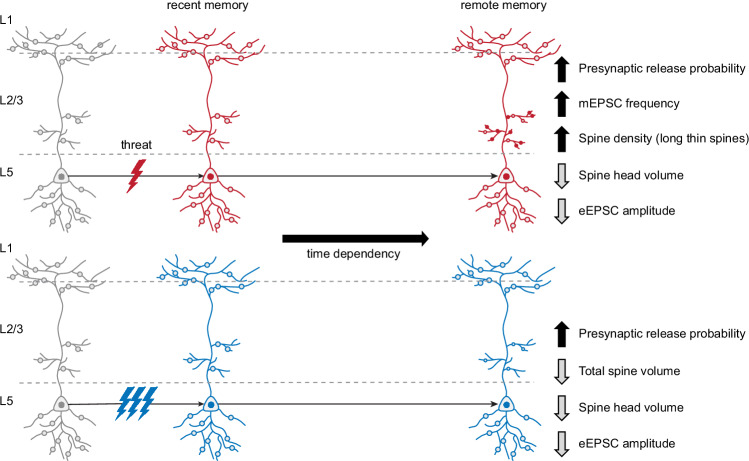
Schematic of the engram architecture of layer 5 PL PNs activated during threat conditioning. Summary of key electrophysiological and structural adaptations. Circles on dendrites represent spines. All neuroadaptations emerge in a time-dependent manner, i.e., they are present at the remote timepoint ( ~ 1 month) after CTC and do not develop yet within the first week after training. In addition, they do not occur in absence of a foot-shock and therefore reflect associative learning during an aversive experience. Adaptations not influenced by threat intensity involve an increase in presynaptic release probability onto CTC-activated PL PNs, accompanied by reduced postsynaptic strength (evoked EPSC amplitude) and spine size. Addition of long thin spines (filled red circles) on oblique dendrites of the apical trunk of engram cells occurs exclusively after mild CTC, which is accompanied by increased miniature EPSC (mEPSC) frequency onto these neurons.

We demonstrate that PL pyramidal engram cells in layer 5 develop physiological and structural synaptic adaptations that are shaped by threat intensity and the passage of time (Fig. 7). These modifications align with their selective recruitment in the expression of a remote mild, but not strong, threat memory. Specifically, we found that enhanced spine density on PL pyramidal engram cells after mild CTC is driven by the addition of long thin spines on their oblique dendrites. We also observed neuroadaptations that are induced by CTC but independent of threat intensity, including enhanced presynaptic release probability onto CTC-activated PL PNs, alongside reduced postsynaptic strength. The latter is likely due to a shift in spine morphology towards smaller spine heads on CTC-activated PL neurons, which was complemented by a decrease in the total spine volume specifically after strong CTC. Thus, these data point to a synaptic signature in which addition of new long thin spines promotes engagement of PL PNs in a mild threat engram. Conversely, the decreased spine size and postsynaptic strength without addition of dendritic spines likely contributes to disengagement of learning-activated PL PNs from a strong threat engram. This is further supported by our finding that the reactivation level of these neurons during remote memory retrieval is lower than the reactivation rate of mild threat-activated PL neurons.

Chemogenetic suppression of CTC-activated PL neurons impairs the expression of mild (1 foot-shock), but not strong (3 foot-shocks), threat memory in TRAP2 mice. This intervention results in a partial reduction of freezing behavior, likely because the PL functions as a hub in a larger engram circuit. Suppression of engram cells in a distributed network involving multiple brain regions, e.g., including the BLA^11^, may be required to fully block memory expression^30^. As the same intervention does not affect expression of a strong threat memory, learning-activated PL neurons do not appear to functionally contribute to the broader, brain-wide engram circuit after a stronger aversive experience. We hypothesize that strong threat memories rely more heavily on evolutionary conserved subcortical brain regions that process emotional stimuli, such as the amygdala, and less on top-down control over these regions by the prefrontal cortex.

Reciprocal connectivity between the mPFC and BLA engram mediates formation and retrieval of remote threat memory^11,12,17,31^. PL-to-BLA projections also suppress generalized threat responses through activation of somatostatin interneurons in the BLA^32^. In line with the latter, somatostatin interneurons are activated during threat conditioning and modulate threat responses through inhibition of excitatory engram cells in the BLA^33^. Hence, PL inputs can alter the activity of the BLA engram by directly innervating excitatory BLA engram cells or indirectly via inhibitory interneurons in this region. Interestingly, whereas the number of PL neurons activated during mild and strong threat conditioning does not differ^10^, the number of activated BLA neurons increases with increasing threat intensity^10,34^. Moreover, excitatory (and inhibitory) synaptic transmission onto BLA engram cells amplifies as a function of conditioning strength^35^, in contrast to our findings in the PL. The differential effect of threat intensity on engram size and synaptic properties in PL compared with BLA could result in reduced PL influence over the BLA after a highly aversive experience, potentially changing the balance of excitatory versus inhibitory synaptic input onto BLA engram cells.

Cortical top-down control enables adaptive behavior, including the suppression of responses to conditioned stimuli that no longer predict a threat^36^. Hence, disengagement of the PL engram from remote strong threat memories may explain the slower extinction learning in mice that undergo strong threat conditioning, similar to humans^37^. Resilience to extinction is also a hallmark of maladaptive memories, such as in PTSD^1^. Of relevance to this is that PTSD patients exhibit hypoactivation of the mPFC in response to aversive stimuli, unlike healthy humans that experienced threat conditioning^13^, suggestive of a similar attenuation of top-down control by the mPFC depending on the intensity of an aversive experience.

Several days after learning, CTC-activated PL pyramidal cells do not differ from neighboring non-activated pyramidal cells in their excitability and synaptic properties. This aligns with the observation that PL engram cells are not yet necessary, nor reactivated, during recent memory expression^10,11^. Together, our findings indicate that cortical engram cells undergo gradual synaptic adaptations to allow their functional engagement in memory expression, in line with theories of memory systems consolidation^8^ and reorganization^38^. The lack of detectable changes at a recent timepoint after CTC contradicts with physiological and structural synaptic adaptations in mPFC neurons 1 h after CTC^39^. However, that study did not specifically focus on engram cells and these rapid changes may reflect short-lived plasticity mechanisms. Our data also contrast with the finding that excitability of layer 5 pyramidal engram cells in the caudal anterior cingulate cortex is transiently enhanced within the first 3 days after CTC^40^. The discrepancy may be explained by the difference in brain region or timepoint (d1-3 in their study vs. d6-7 in our study) at which cells were recorded. Transient changes induced by CTC may no longer be detectable several days after learning. Although transient, such early processes may serve as a critical trigger for initiating a lasting molecular program in cortical engram cells to support their gradual functional integration into a remote memory engram circuit^10,41^.

Spine density increases on PL PNs at a remote timepoint exclusively after mild CTC and this is corroborated by an increase in mEPSC frequency. These findings extend previously reported time-dependent increases in spine density on cortical neurons after CTC^11,42^, emphasizing the importance of non-Hebbian synaptic plasticity in engram persistence^43,44^. We further show that the enhanced spine density reflects the addition of long thin spines on oblique dendrites of layer 5 PL pyramidal engram cells. Notably, a similar increase in the proportion of long thin spines versus mushroom spines, has been reported on mPFC PNs 1 h after CTC^39^. Mushroom spines are considered memory-type spines owing to their stability, large spine head and abundance of AMPA receptors, whereas long thin spines function as learning-type spines due to their transient nature, smaller spine head and fewer AMPA receptors^23^. Consequently, long thin spines represent weaker synapses than mushroom spines. However, they exhibit enhanced capacity to undergo activity-dependent adaptation of their spine head and recruit or remove AMPARs, thereby enabling a synapse to strengthen or weaken, as such being highly plastic^45^. In the amygdala, small spines, including long thin spines, are preferentially contacted by cortical afferents, whereas larger spines receive thalamic input^46^. To our knowledge, it is unknown whether the same heterogeneity applies to cortical spines, but if similar principles hold, the selective addition of long thin spines on PL engram cells may point to a enhanced intracortical connectivity. Interestingly, long thin spines in the prefrontal cortex are enriched in proteins involved in calcium-cAMP signaling, which facilitates gating of synaptic input to dendrites, thereby contributing to flexibility in network strength^47^. We speculate that enhanced synaptic connectivity of PL engram cells by addition of long thin spines on their oblique dendrites may facilitate synaptic plasticity, rendering memories formed under mild threat conditions more adaptable over time.

Although presynaptic release probability onto CTC-activated PNs is enhanced irrespective of threat intensity, the combination of augmented presynaptic transmission and postsynaptic spine density after a mild threat may be required to drive a persistent afferent-specific enhancement of connectivity at the apical trunk of layer 5 pyramidal engram cells, i.e., involving inputs that innervate layer 2/3 of the PL (Fig. 7)^16^. Potential sources that innervate layer 2/3 and have been implicated in remote threat memory are the contralateral PL^12,19^ and basolateral amygdala^11,19^. Furthermore, nuclei of the medial dorsal thalamus project to layer 2/3 of the PL^18,48^ and are thought to have a role in memory systems consolidation^20^. Alternatively, the increase in presynaptic release probability may result from homeostatic synaptic plasticity^49^, such that the reduction in postsynaptic strength (through decreased eEPSC amplitude and smaller spines) may be compensated by an increase in the probability of presynaptic vesicle release to maintain synaptic weigth^50^. Whether the increased presynaptic release probability serves to strengthen connectivity of PL engrams cells with one or more input regions in a threat intensity-dependent manner, or to compensate for the reduced postsynaptic strength, remains an important topic for future research.

Paradoxically, the increased spine density on PL engram cells is accompanied by the reduction in eEPSC amplitude in the same neurons, which developed after both mild and strong CTC. As AMPAR-NMDAR current ratios were unaffected in PNs activated during CTC, signaling through both glutamate receptors is likely equally reduced. This decrease in postsynaptic strength is supported by the reduction in spine head volume on tagged PL neurons after mild and strong CTC^23^. Our findings suggests memory systems consolidation by cortical engram cells involves heterosynaptic plasticity, in line with computational models of systems consolidation of memory^44^. Strengthening of specific afferent connectivity, e.g., between PL engram cells^12^, may be balanced by decreased postsynaptic strength, potentially occurring in nearby synapses not engaged in the threat memory trace. This might preserve homeostasis of synaptic weight within an engram cell. Strengthening of a small subset of synapses combined with weakening of other synapses would explain why we did not detect a change in the overall AMPA/NMDA ratio.

We report shared, as well as unique, electrophysiological and structural adaptations in tagged PNs after a mild and strong aversive experience, which do not develop when mice are not exposed to an aversive stimulus (No US). However, our study has some limitations. Firstly, we specifically focussed on layer 5 PNs. Given that PNs in superficial (layer 2/3) and deep (layer 5/6) layers differ in physiological and morphological properties, as well as their connectivity^51^, it is possible that CTC induces changes in layer 2/3 PNs that are distinct from those in layer 5. Secondly, due to the methodological design of our electrophysiological experiments, we did not directly compare PN populations between conditions (see Methods). Therefore, our conclusions are based only on differences between tagged and non-tagged populations within an experimental condition. Nonetheless, such differences may be required to increase the signal-to-noise ratio of tagged versus non-tagged PNs within the local network, as supported by differences in the reactivation rate of tagged neurons during retrieval of a mild versus strong threat memory.

To conclude, we reveal that neuroadaptations in layer 5 PL pyramidal engram cells exhibit three key characteristics: they depend on (1) threat intensity, (2) the passage of time, and (3) develop in a dendritic segment-specific manner. As such, our findings provide strong support for (1) the functional engagement of PL engram cells in expression of a mild threat memory, (2) their contribution to remote, but not recent, memory expression^10,11^, and (3) increased potential for enhanced connectivity with afferents innervating layer 2/3 of the PL. Hence, the data underscores that memory representations in the brain are shaped by the intensity of aversive experiences. This has important implications for understanding the strength and persistence of threat memories, and potentially how adaptive versus maladaptive threat memories, such as those in PTSD, arise through differential engram circuits.

## Methods

### Animals

Fos^2A-iCreER/+^ (TRAP2) (stock #030323) and R26^AI14/+^ (AI14) (stock #007914) mouse lines were obtained from the Jackson Laboratory. TRAP2 mice were crossed with AI14 mice to acquire the double heterozygous (TRAP2;Ai14) used in this study^15^. Male mice aged 8-10 weeks at the start of the experiment were housed individually on a 12-h light/dark cycle with food and water available *ad libitum*. Ambient temperature and humidity were controlled between 19 °C to 23 °C and 40% to 70%, respectively. Behavioral experiments were performed during the animals’ light phase. Independent cohorts were used for experiments in Fig. 1 (and distinct groups for panels a-c, d-f and g-i), as well as the electrophysiological experiments in Figs. 2,3 and 5. Spine density quantifications (Fig. 4) were performed on neurons that were filled with biocytin during eEPSC recordings (Fig. 3). Spine reconstruction and classification (Fig. 6) were performed on neurons that were filled with biocytin during mEPSC recordings (Fig. 5). All experimental procedures were approved by the Central Committee for Animal experiments (Centrale Commissie Dierproeven) of The Netherlands and the animal ethical care committee (Instantie voor Dierenwelzijn) of the Vrije Universiteit Amsterdam.

### AAV vectors and stereotactic micro-injections

AAV-*hSyn*::DIO-mCherry and AAV-*hSyn*::DIO-hM4Di-mCherry (titers: 5.0-6.0 ×10^12^) were packaged as serotype 2/5. For stereotaxic micro-injection of AAVs, mice received 0.05 mg/kg Temgesic (RB Pharmaceuticals, UK) 30 min before the start of surgery, were then anesthetized with isoflurane and mounted onto a stereotactic frame. Lidocaine (2%, Sigma-Aldrich Chemie N.V., The Netherlands) was topically applied to the skull before incision to provide local analgesia. Microinjection glass needles were used to infuse virus in the mPFC ( + 1.9 mm AP; +0.45 mm ML; -2.1 mm DV; relative to Bregma) at a flow rate of 0.1 µL/min followed by an additional 5 min to allow diffusion, followed by stepwise retraction of the needle. For chemogenetic suppression of CTC-tagged neurons, Cre-dependent AAV-*hSyn*::DIO-hM4Di-mCherry or AAV-*hSyn*::DIO-mCherry virus (0.5 µL/hemisphere) was bilaterally injected in the PL of TRAP2 mice. Analgesia was provided 24 h before and up to 48 h after surgery (Carprofen, 5 mg/kg). Animals remained in their home-cage for 3 weeks until the start of behavioral experiments.

### Contextual Threat Conditioning

Mice were handled for two successive days and after an interval of 48 h, underwent contextual threat conditioning (CTC)^10^. Conditioning was performed in a Plexiglas chamber with a stainless-steel grid floor inside a soundproof cabinet with continuous white noise (68 dB; Ugo Basile, Italy). The chamber was cleaned with 70% ethanol in between the trials. Mice were allowed exploration of the context for 120 s prior to the onset of an unconditioned stimulus (US): 2 s long 0.7 mA foot-shock (1US CTC). For 3US CTC, mice received three foot-shocks with an interval of 60 seconds. All mice were returned to their home-cage 30 seconds after the last foot-shock. Mice in the context control group (No US) explored the CTC box for 150 s in absence of a foot-shock. During a memory retrieval session, mice were allowed to explore the CTC context for 2 min. For the extinction session, context exploration was allowed for 15 min. Freezing behavior was recorded with a video camera and analyzed using the Ethovision XT video-tracking software (Noldus, The Netherlands). Freezing bouts were defined as a lack of movement excluding breathing for a minimum of 1.5 s.

### 4-OHT treatment

First, 3 mg of 4-Hydroxytamoxifen (4-OHT; H6278, Sigma-Aldrich Chemie N.V., Netherlands) was dissolved in 60 μL of 100% dimethyl sulfoxide (DMSO) (D8418, Sigma-Aldrich Chemie N.V, The Netherlands). This stock solution was stepwise-diluted in 570 μl of saline containing 2% Tween 80 (P1754, Sigma-Aldrich Chemie N.V., Netherlands) and then once more in the same volume of saline. The final solution consisted of 2.5 mg/ml 4-OHT, 5% DMSO, and 1% Tween80 in saline^52^. Animals received 4-OHT (25 mg/kg, i.p.) 1 h after a behavioral tag session.

### Immunohistochemistry

Mice were transcardially perfused using ice-cold phosphate-buffered saline (PBS) pH 7.4, followed by ice-cold 4% paraformaldehyde (PFA) in PBS pH 7.4. Brains were removed, post-fixed overnight in 4% PFA solution and then immersed in 30% sucrose in PBS with 0.02% NaN_3_. Brains were then sliced in 35 μm coronal sections using a cryostat and stored in PBS with 0.02% NaN_3_ at 4 °C until further use. Immunohistochemical stainings were performed using standard procedures using the NeuroTrace^TM^ 500/525 Green Fluorescent Nissl Stain (Thermo Fisher Scientific) or DAPI stain. For Fos quantification, slices were stained using primary rat anti-Fos antibody (SySy, #226017, 1:1000) and secondary goat anti-rat Alexa 647 antibody (Life technologies, A21247, 1:400). For quantification experiments, 4-6 z-stacks per animal were generated using a confocal microscope (Zeiss LSM510 or Nikon A1R) with the experimenter blind to the treatment conditions. ImageJ software was used to extract the ROIs of the cells stained with Nissl or DAPI (Gaussian filter, Li threshold). Only ROIs within a predefined range for size (70-2000 square units) and circularity (0.5 to 1.0) were included to exclude glial cells and non-specific staining. As cells were frequently present in 2 or 3 subsequent images of a z-stack, MATLAB (MathWorks) was used to group the ROIs that belonged to the same Nissl cell, and then the total number of Nissl^+^ cells in a stack were quantified. Cells expressing tdTom and/or Fos were counted manually.

### Chemogenetic intervention

Clozapine N-oxide (CNO; HB6149 HelloBio, United Kingdom) was dissolved in sterile saline. Mice received an injection of 5 mg/kg (i.p.) CNO 30 min before a retrieval session.

### Acute brain slice preparation

Electrophysiological recordings were performed at two different timepoints, 3–7 days after behavior (recent timepoint), or 28-31 days later (remote timepoint). Ex vivo brain slices were prepared as previously described^53^. Mice were anaesthetized (Avertin, i.p., 250 mg/kg) and transcardially perfused using a previously carbogenated (95% O2, 5% CO2) ice-cold partial sucrose solution containing (in mM): 70 NaCl, 2.5 KCl, 1.25 NaH_2_PO_4_*H_2_0, 5 MgSO_4_*7H_2_O, 1 CaCl_2_*2H_2_O, 70 Sucrose, 25 D-Glucose, 25 NaHCO_3_, 1 Na-Ascorbate, 3 Na-Pyruvate ( ~ 300 mOsm/kg, pH 7.4). Brains were carefully extracted, and the frontal half trimmed and glued onto the stage of a vibrating microtome (Leica, VT1200). Coronal slices (thickness 300 µm) containing the PL were cut in the carbogenated ice-cold partial sucrose solution. Slices were then transferred to continuously carbogenated holding artificial cerebrospinal fluid (aCSF) containing (in mM): 125 NaCl, 3 KCl, 1.25 NaH_2_PO_4_*H_2_O, 2 MgCl_2_*6H_2_O, 1.3 CaCl_2_*2H_2_O, 25 D-Glucose, 25 NaHCO_3_, 25 D-Glucose, 25 NaHCO_3_, 1 Na-Ascorbate, 3 Na-Pyruvate ( ~ 300 mOsm/kg, pH 7.4), where they would recover for at least one hour at room temperature prior to the recording.

### Electrophysiological recordings and analysis

Sections were transferred to a submerged recording chamber of an upright microscope (BX51WI, Olympus) and continuously perfused (2 mL/min, 32 °C) with carbogenated running aCSF (holding aCSF without Na-Ascorbate, Na-Pyruvate and only 1 mM MgCl2*6H2O). Under visual guidance of differential interference contrast microscopy, the PL was visualized, and layer 5 PNs were identified based on their distance from the midline ( ~ 300–500 µm), pyramidal like shape, large size of the soma and presence of thick apical dendrites directed towards the midline. Tagged (tdTom^+^) PNs were visualized based on somatic and dendritic tdTom expression using a set of filters (DMLP567R, MF630-69, ThorLabs) in combination with 625 nm excitation wavelength (LED4D251, ThorLabs). Non-tagged (tdTom^-^) PNs in close proximity of tdTom^+^ PNs in the PL were recorded. Patch pipettes (3–5 MΩ resistance) were pulled from borosilicate glass capillaries (Science Products) using a horizontal micropipette puller (P-87, Sutter Instruments Co.). The pipette was mounted on a headstage of a Multiclamp 700B amplifier (Molecular Devices). 1US CTC excitability measurements were acquired with pClamp software (Molecular Devices) and a Multiclamp 700B amplifier (Molecular Devices), sampled at 20 kHz, low-pass filtered at 6 kHz, and digitized with the Axon Digidata 1440 A (Molecular Devices). All other recordings were low-pass filtered at 4 kHz and digitized at 50 kHz using a USB-6343 (National Instruments), wherein all of the commands were generated, signals processed, and amplifier metadata acquired using MIES, written in Igor Pro (Wavemetrics). Prior to whole-cell access, a giga-ohm seal would be formed. Cells were allowed ~5 min for stabilization of the patch after break-in, and recordings were included in the analysis if Ra was less than 20 MΩ and would not change more than 20% for the duration of the recording. Access resistance (Ra) and membrane capacitance (Cm) were calculated in the voltage-clamp configuration by analyzing capacitive transients during 10 ms square wave -10 mV pulses.

### Excitability measurements

Excitability was assessed using potassium gluconate-based intracellular solution containing (in mM): 148 K-Gluconate, 1 KCl, 10 Hepes, 0.3 EGTA, 4 Mg-ATP, 4 K2-phosphocreatinine, 0.4 GTP (sodium salt), osmolarity 280-290 mOsm/kg, pH adjusted to 7.3 with KOH. Synaptic transmission was not blocked during excitability recordings. Passive membrane properties, action potential (AP) shape and AP firing rate were determined as previously described^54^. Recordings were performed at resting membrane potential (RMP) which was determined upon stabilization of the patch as median of a 1 min 0 pA current-clamp recording. Depolarizing square pulses (50 ms) with a 5 pA increment were applied at 1 Hz, and *Rheobase* was the minimum amount of current to induce the first AP. All AP properties (except for medium afterhyperpolarization (mAHP)) were extracted from five consecutive APs induced by a 50 ms Rheobase +10 pA depolarizing current. The first derivative was used to extract the maximum speed of depolarization (dv/dt_max_) and repolarization (dv/dt_min_). AP threshold was determined where the depolarization speed first exceeded 10 mV/ms, and fast AHP (fAHP) within 3 ms from the AP peak. AP amplitude and AHPs are relative to the AP threshold.

To extract additional biophysical parameters depolarizing current steps (750 ms, 20 pA increment) were applied every 3 s from −100 pA to 500 pA. Input resistance was calculated as the linear slope of the sustained response and hyperpolarizing steps. Membrane time constant was obtained by a single exponential fit of the initial 80 ms of the voltage response to a − 80 pA current injection. Sag ratio (%) was calculated from the hyperpolarization step ~ −7.5 mV as (V_peak_ - V_sus_)/(V_peak_), with V_peak_ as the peak hyperpolarizing response relative to baseline and V_sus_ as the sustained response last 100 ms of the current step relative to baseline. mAHP was measured from the 2^nd^ to the 4^th^ AP (15-100 ms from the AP peak) of the first depolarizing step with >4APs. To generate the input–AP output, instantaneous AP frequency (1/interspike interval (ISI)) was averaged and plotted against the depolarizing current step. Adaptation ratio (1^st^ ISI/9^th^ ISI) was calculated from the first step with at least 10 APs.

### Synaptic recordings

Synaptic properties were assessed using the caesium gluconate-based intracellular solution containing (in mM): 130 Cs-Gluconate, 8 NaCl, 10 Hepes, 0.3 EGTA, 4 Mg-ATP, 10 K2-phosphocreatinine, 0.3 GTP (sodium salt), 3 QX134-Cl, osmolarity 280-290 mOsm/kg, pH adjusted to 7.3 with CsOH. Furthermore, inhibitory synaptic transmission was blocked by extracellular 10 μM gabazine, a GABA-A receptor antagonist (HelloBio, HB0901).

Evoked excitatory postsynaptic currents (eEPSC) were recorded in the presence of 10 µM glycine, a necessary cofactor for NMDAR activation. Stimulation electrode was placed in cortical layer 2–3, 50–100 µm laterally to the recording electrode, and repositioned until a clear unisynaptic response was observed in response to current injections (Master-9 pulse stimulator and ISO-flex stimulus isolator, A.M.P.I.). Thereafter, an input-output stimulation curve was generated in response to increasing amounts of current ( + 2 µA, 0.1 Hz). For the AMPA/NMDA ratio and paired pulse ratio (PPR) recordings, we used the stimulation intensity corresponding to the half-maximal eEPSC amplitude in the input-output curve. EPSCs were averaged from 10–30 sweeps sampled at 0.1 Hz. AMPAR-mediated responses were obtained as the peak inward current at −70 mV. NMDAR-mediated responses were measured at 40 mV, corrected by the response that remained at 0 mV and calculated as the mean outward current between 45 and 55 ms after the stimulus^55^. The interstimulus intervals for the PPR protocol were 50 ms and 100 ms.

In order to record miniature EPSCs (mEPSCs), action potential-dependent neurotransmission was blocked by adding 0.75 μM tetrodotoxin-citrate (HelloBio, HB1035) to the extracellular solution. Upon achieving a stable whole-cell configuration, mEPSCs were recorded gap-free for up to 10 min at −70 mV, out of which only the last 5 min were included in the analysis. mEPSCs were analyzed with the MiniAnalysis software (Synaptosoft) and automatically detected using the following parameters: threshold 7 pA, period to search a local maximum 20 ms, time before a peak for baseline 8 ms, period to search a decay time 20 ms, fraction of peak to find a decay time 0.37, period to average a baseline 2 ms, area threshold 20 pA*ms, number of points to average a peak 3, direction of peak negative. False positives and false negatives in automatically detected events were checked manually by an experimenter blinded to the conditions. For all events, amplitude and inter-event interval (expressed as frequency) were noted and either averaged per neuron or a cumulative distribution plot was generated by randomly sampling 100 events per neuron. Signal processing and data analysis was performed using custom-written Matlab (Mathworks) or RStudio scripts.

### Structural analyses

For the duration of patch-clamp recordings, a subset of neurons was filled with biocytin. Upon completion of the recording the pipette was slowly retracted to reseal the patched cell, and the slice was subsequently fixed in 4% PFA, at 4 °C for 24−48 h. For long term storage, slices were stored in PBS with 0.02% NaN_3_. Slices containing biocytin filled cells were incubated in the blocking solution (3% normal goat serum, 1% BSA, 0.5% Triton™ X-100 in 1x PBS) for 2 h at RT, and then for 24 h at 4 °C in primary antibody anti-RFP (1:2000, Tebu-Bio, Article Number 600−401-379) in blocking solution. Subsequently, slices were incubated 24 h at 4 °C with secondary antibody goat anti-rabbit, Alexa Fluor-633 (1:400, Molecular Probes, A21070) and streptavidin conjugated to Alexa Fluor−488 (1:500, Molecular Probes, S-11223/S325). Lastly, DAPI nuclear staining was performed. All steps were separated by 2−4x washes with PBS. Anti-fading mounting medium (Sigma Aldrich, PVADABCO^®^) was used for coverslipping.

#### Spine density analysis

For data in Fig. 4, imaging of dendritic spines and analysis was performed in a subset of eEPSC recorded neurons. Neurons were imaged using a confocal microscope (NIKON, Eclipse Ti A1). Identity of tdTom^+^ neurons was confirmed based on the colocalization of conjugated streptavidin and tdTom. Neurons with sufficiently filled dendrites and clearly visible spines were included in the analysis. The 2^nd^ and 3^rd^ order dendrites were imaged and based on their dendritic segment separated into basal, apical trunk or apical tuft dendrites. Z-stacks were obtained of dendritic fragments (length ~ 30–50 μm) with a 63x oil-immersed objective. Images were analyzed and spines were counted using Fiji software (https://fiji.sc/). Spine density was determined by dividing the number of spines by the length of the imaged dendrite and was averaged per compartment per neuron.

#### Spine morphology analysis

For data in Fig. 6, images of biocytin filled cells that were recorded for mEPSCs were obtained using a confocal microscope (NIKON, Eclipse Ti A1) and a 40x oil-immersion objective. A 6.21x times zoom was used to obtain a pixel size of 0.05 µm/pixel. Z-steps of 0.16 µm were used in agreement with optimal imaging settings according to Nyquist calculations. Per neuron, 4–6 images of oblique dendrite fragments at the apical trunk were obtained. Dendrites and spines were traced using IMARIS 9.9.1 software in a semi-automated manner. The dendrite was manually traced with automated center detection. The dendrite diameter was set to a threshold of 0.05 based on local contrast. Thinnest spine diameter and maximal spine length were set to 0.1 µm and 5 µm, respectively. Seed points for spine detection were set at an automated threshold and then manually edited to obtain one seed point per spine to improve tracing. Spine diameter threshold was set to 0.05 based on local contrast. For each image, all spine measurements were exported and further analyzed using MATLAB. Then, spines were classified based on their length and ratio between volume of the head and volume of the neck. Spines were classified as stubby if they had a length <1 µm and a head-neck volume ratio <1.2. If a spine did not contain a neck it was also classified as stubby. If a spine was >1 µm in length and had a head-neck volume ratio <1.2, it would be classified as long thin. Mushroom spines were classified as having a head-neck volume ratio >1.2 without criteria for length. Spines that did not fit these criteria (*n* = 4) were excluded from the analysis. Density per spine type was calculated by dividing the total number of spines of a subtype detected on all dendritic fragments by the total length of the traced dendritic fragments per neuron.

### Quantification and statistical analysis

Statistical details are presented in the figure legends and Supplementary Table 3. Prism 9 (Graphpad) and RStudio were used for statistical analysis. For between group comparisons, two-tailed unpaired Student’s *t*-test or a Mann–Whitney *U*-test was used, depending on whether the data followed a normal distribution. For electrophysiological analyses, we recorded cells from 1 mouse per day. To reduce the impact of small day-to-day differences in slice conditions, recording variables and placement of the stimulating electrode, statistical analyses were limited to within-subject pairwise comparisons of tagged and non-tagged neurons per experimental condition. A one-tailed Mann–Whitney *U*-test was used for mEPSC frequency comparison after 1US CTC based on the assumption that mEPSC frequency was increased as a result of the increase in spine density after 1US CTC. Kolmogorov–Smirnov and Shapiro–Wilk tests were used to assess normality. In case of normally distributed data with unequal variances, Welch’s unequal variance *t*-test was used. For cumulative distributions, the Kolmogorov-Smirnov test was used for between group comparisons. In case of comparisons that involved more than 2 groups, one-way ANOVA was performed followed by post-hoc Bonferroni test. In case of more than 1 within-subject comparison, two-way repeated measures (RM) ANOVA or Mixed-effects model (in case of missing values) was carried out with Population (tdTom^-^ vs. tdTom^+^) or Group (1US vs. 3US) as between-subject factor and Stimulation (e.g., AP input-output, eEPSC input-output), ISI (PPR) or time (extinction) as repeated measures. Post-hoc Bonferroni test was performed in case of between-subject comparisons for more than 1 repeated measures. Outliers were determined using the Grubb’s test. One mouse from the 1US CTC mCherry behavioral data set was removed as an outlier, and 2 mice from the 3US CTC hM4Di behavioral dataset was removed due to virus misplacement. For Supplementary Fig 1, we excluded 3 mice from the 1US group that froze less than 15% during the first two minutes of the extinction session, as their freezing levels were too low to detect extinction learning. For comparisons of cumulative distributions, significance was set at *p* < 0.001 to correct for high sample size, otherwise significance was set at *p* < 0.05. For analyses of 3D reconstructed dendrites and spines, hierarchical multilevel regression models with random intercepts were built for each dependent variable as previously described^28^. For spine density measures, the tested random intercepts included (mouse, slice, cell, mouse/slice, slice/cell, mouse/slice/cell) and for spine morphology measures (mouse, slice, cell, dendrite, mouse/slice, slice/dendrite, slice/cell, mouse/slice/cell, slice/cell/dendrite, mouse/slice/cell/dendrite), respectively. Violin plots display data distribution of overall spine morphology parameters and individual data points are colored according to the random intercept. Bar graphs of spine densities display mean and s.e.m., as well as individual data points colored according to the random intercept.

### Reporting summary

Further information on research design is available in the Nature Portfolio Reporting Summary linked to this article.

## Supplementary information

Supplementary information

Reporting Summary

Transparent Peer Review file

## Source data

Source Data

## Data Availability

All behavioral, cellular, electrophysiological and structural data generated in this study are provided in the Source Data file. Source data are provided with this paper.

## Code availability

Code used for spine analysis is available on Github(https://github.com/PantheaNemat/Mitric-Beerens_etal_2026_Nat.Commun.) and Zenodo (10.5281/zenodo.19483826).
