# Supplementary material for: Threat intensity shapes cortical engram architecture supporting remote memory retrieval
Source: Nat Commun. 2026 Jun 11;17:7447. doi: 10.1038/s41467-026-74231-5 (PMC13408494; doi:10.1038/s41467-026-74231-5)
Supplement: Supplementary file 1 — Supplementary information [file 41467_2026_74231_MOESM1_ESM.pdf]

Supplementary information

Threat intensity shapes cortical engram architecture supporting remote memory retrieval

Authors

Miodrag M. Mitrić[1,#], Sanne Beerens[1,#], Panthea Nemat[1], Esther Visser[1], Luca van Leeuwen[1], Rolinka J. van der Loo[1], August B. Smit[1], Priyanka Rao-Ruiz[1,*] and Michel C. van den Oever[1,*]

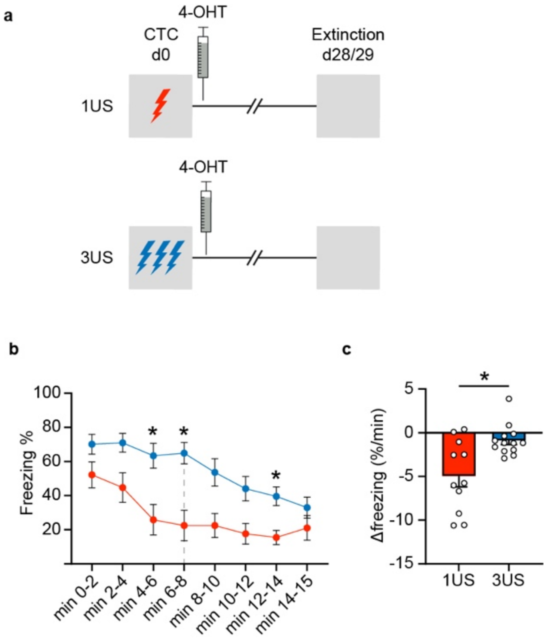

Supplementary Figure 1. Strong threat memory is more resilient to extinction learning than mild threat memory. a Experimental design. Mice were exposed to either 1US or 3US CTC. At day 28/29 after CTC, mice were alowed to freely explore the conditioning context for 15 min in absence of foot-shocks. **b** Freezing levels did not differ significantly during the first 2 min of the session, but subsequently extinguished faster in the 1US group. Two way RM ANOVA: Time x Group: $F_{7,154}$ = 2.2, p = 0.028, Group: $F_{1,22}$ = 12.3, p = 0.002, Time: $F_{7,154}$ = 16.2, p < 0.0001). Post-hoc. Bonferroni test revealed a difference between groups at min 4-6 (*p = 0.032), min 6-8 (*p = 0.008) and min 12-14 (*p = 0.016). 1US: n = 11, 3US: n = 13. Grey dashed line indicates the middle of the extinction session. **c** Given that 1US mice already reached stable low freezing levels halfway the session, we compared the change in freezing between min 0-2 and min 6-8 by calculating the slope. This also confirmed that 1US mice extinguished faster than 3US mice (Mann-Whitney U test, U = 34, p = 0.030). All statistical analyses were performed two-sided. Bar graphs show mean + s.e.m. Individual data points are mice. Source data are provided as a Source Data file.

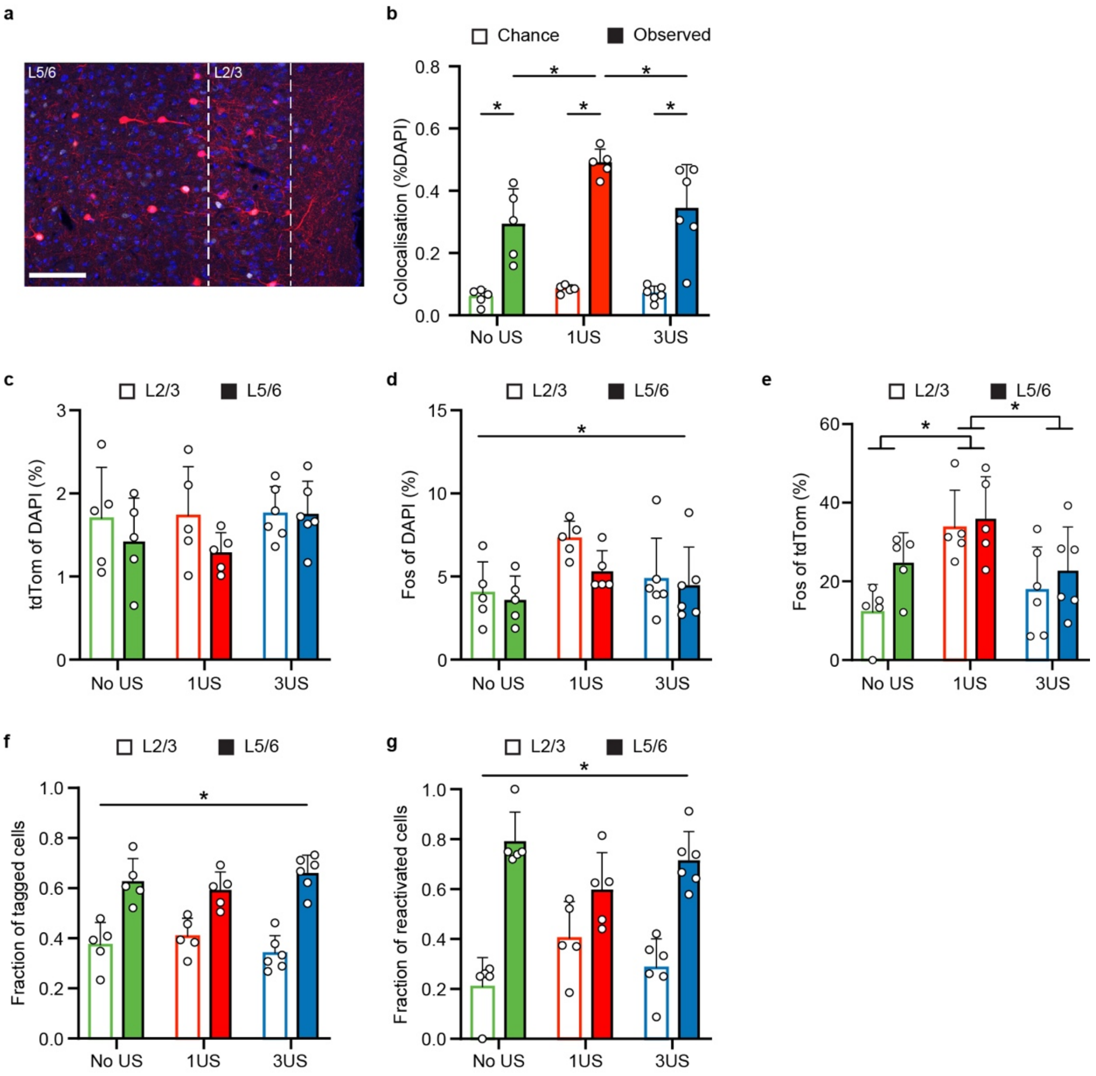

Supplementary Figure 2. Tagging and reactivation across superficial and deep layers in the PL. Data is derived from groups presented in Fig.1g. **a** Representative image from Fig. 1h. Example of segmentation of the PL into superficial (L2/3) and deep (L5/6) layers. **b** With all layers combined, colocalization of Fos and tdTom was higher than chance level across all groups, with the 1US CTC group having a higher observed colocalization level than the No US and 3US CTC groups (RM ANOVA: Group: $F_{2,13} = 4.0$, $p = 0.04$, Observed/chance: $F_{1,13} = 164.8$, $p < 0.0001$, Group x Observed/chance: $F_{2,13} = 4.6$, $p = 0.03$; post-hoc Tukey test: No US observed vs. No US chance $p < 0.0001$, 1US observed vs. 1US chance $p < 0.001$, 3US observed vs. 3US chance $p < 0.001$, No US observed vs. 1US observed $p = 0.002$, No US observed vs. 3US observed $p = 0.67$, 1US observed vs. 3US observed $p = 0.02$). **c** Percentage of $tdTom^+$ neurons did not differ across layers or groups (RM ANOVA: Group: $F_{2,13} = 0.6$, $p = 0.56$, Layer: $F_{1,13} = 3.95$, $p = 0.07$, Group x Layer: $F_{2,13}=1.19$, $p = 0.33$) **d** Percentage of Fos^+ neurons did not differ between groups, but was slightly higher in superficial layers compared with deep layers (RM ANOVA: Group: $F_{2,13} = 2.9$, $p = 0.09$; Layer: $F_{1,13} = 5.0$, $p = 0.042$; Group x Layer: $F_{2,13} = 1.7$, $p = 0.23$). However, post hoc analyses did not reveal significant differences

between layers within groups. No US $p = 0.93$, 1US CTC $p = 0.052$, 3US CTC $p = 0.94$. **e** With the PL divided into superficial and deep layers, the reactivation rate remained highest in the 1US CTC group, without differences between layers (RM ANOVA: Group: $F_{2,13} = 7.39$, $p = 0.007$, Layer: $F_{1,13}=4.37$, $p = 0.06$, Group x Layer: $F_{2,13} = 0.90$, $p = 0.43$; post-hoc Tukey test: NS vs 1US p=0.01, NS vs 3US p=0.97, 1US vs 3US p=0.02). **f** Of all tagged PL neurons, the fraction of tagged cells is highest in L5/6, without differences between groups (RM ANOVA: Group: $F_{2,13} < 0.001$, $p > 0.99$, Layer: $F_{1,13} = 42.30$, $p < 0.001$, Group x Layer: $F_{2,13} = 1.033$, $p = 0.38$). **g** Of all reactivated neurons, the fraction of reactivated neurons is higher in L5/6 than L2/3, without differences between groups (RM ANOVA: Group: $F_{2,13} < 0.001$, $p > 0.99$, Layer: $F_{1,13} = 40.0$, $p < 0.001$, Group x Layer: $F_{2,13} = 3.0$, $p = 0.09$). All statistical analyses were performed two-sided. Bar graphs show mean + s.e.m. Individual data points are mice. Source data are provided as a Source Data file.

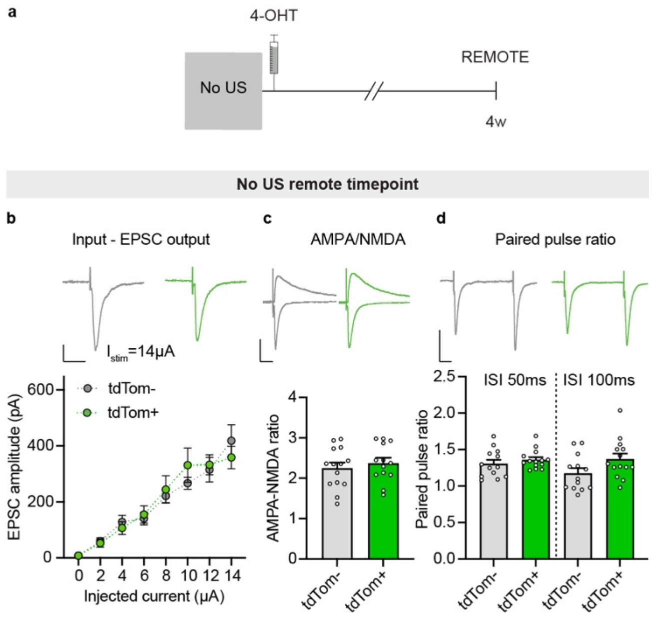

Supplementary Figure 3. Evoked excitatory synaptic transmission is unaltered in mice that undergo context exposure alone. a Experimental design. PL neurons were tagged after exposure to the conditioning context in absence of a foot shock (No US) and 28-31 days later (remote timepoint) eEPSCs were recorded from neighboring $tdTom^+$ and $tdTom^-$ PNs in layer 5. **b** Top: representative eEPSC in response to 14 µA stimulation. The eEPSC amplitude in relation to the stimulation intensity did not differ between $tdTom^-$ (grey) and $tdTom^+$ (green) neurons (Mixed-effects model: Population x Stimulation interaction: $F_{7,180}$ = 0.9, p = 0.52; Population: $F_{1,27}$ = 0.1, p = 0.76). tdTom- *N/n* = 14/7, tdTom+ *N/n* = 15/7. **c** Top: Representative eEPSCs recorded at -70mV (bottom trace) and 40mV (top trace) to determine AMPAR and NMDAR current amplitudes, respectively. Bottom: AMPAR/NMDAR current ratios were similar in $tdTom^+$ and $tdTom^-$ neurons (Unpaired t-test: t_{25} = 0.7, p = 0.51). $tdTom^-$ *N/n* = 14/7, $tdTom^+$ *N/n* = 13/7). **d** Top: representative trace of a PPR recording with 100 ms ISI. PPR showed a trend towards an interaction effect, but did not reach significance (Population x ISI: $F_{1,25}$ = 3.9, p = 0.06; Population: $F_{1,25}$ = 2.8, p = 0.10). tdTom- *N/n*=13/7, tdTom+ *N/n*=14/7. All statistical analyses were performed two-sided. Bar graphs show mean + s.e.m. Individual data points are neurons. Color coding of representative traces matches the bar graphs. Source data are provided as a Source Data file.

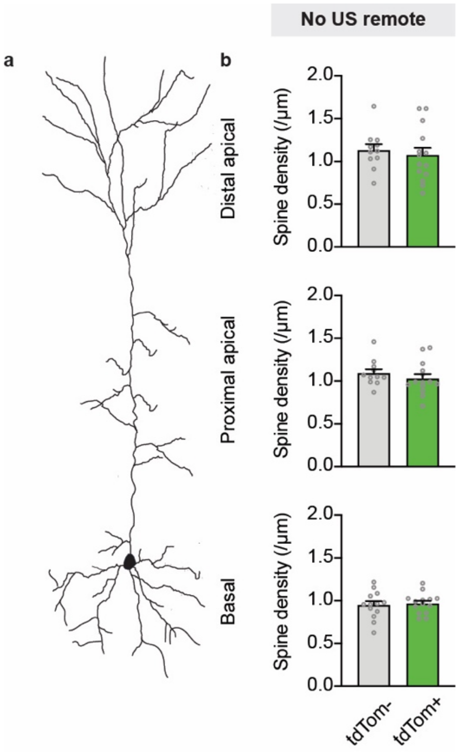

Supplementary Figure 4. Spine density analysis in mice that experienced context exposure alone. Neurons used in eEPSC recordings were filled with biocytin for post hoc spine density analysis. **a** Schematic of a layer 5 pyramidal cell with apical tuft, apical trunk and basal dendrites. **b** Spine density did not differ between tdTom$^+$ and tdTom$^-$ neurons at any dendritic segment (Mixed effects model: Population $F_{1,26} = 0.3$, $p = 0.60$; Population x Segment $F_{2,46} = 0.3$, $p = 0.77$). Tuft: tdTom+, N/n=11/7, tdTom-, N/n=14/6; Trunk: tdTom+, N/n=14/7, tdTom-, N/n=11/7; Basal: tdTom+, N/n=14/7, tdTom-, N/n=13/7. All statistical analyses were performed two-sided. Bar graphs show mean + s.e.m. Individual data points are neurons. Source data are provided as a Source Data file.

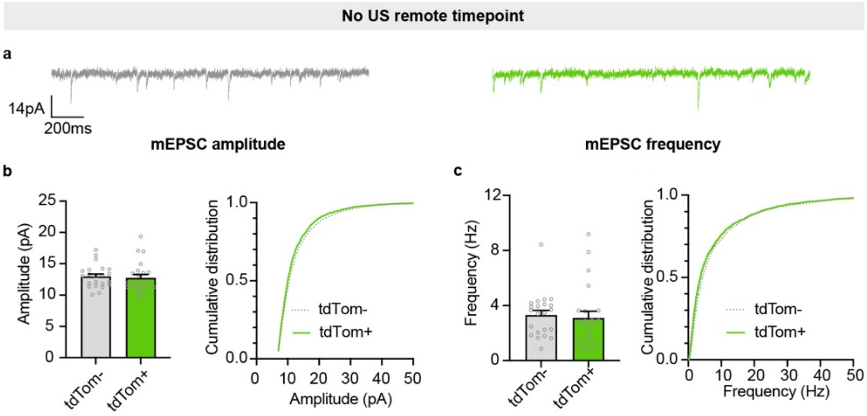

Supplementary Figure 5. Spontaneous excitatory transmission is unaltered in mice that underwent context exposure alone. PL neurons were tagged after exposure to the conditioning context in absence of a foot shock (No US) and mEPSCs were recorded from neighboring tdTom$^+$ and tdTom$^-$ layer 5 PNs 28-31 days later (remote timepoint). **a** Representative traces of mEPSC recordings from tdTom$^-$ (grey) and tdTom$^+$ (green) PNs. tdTom$^-$ N/n = 22/7, tdTom$^+$ N/n = 22/7. **b** Left: the mean mEPSC amplitude did not differ between populations (Mann-Whitney U test: $U = 206$, $p = 0.41$). Right: cumulative distribution plot of mEPSC amplitudes. **c** Left: the mean mEPSC frequency in tdTom$^-$ and tdTom$^+$ neurons did not differ (Mann-Whitney U test: $U = 187$, $p = 0.20$). Right: cumulative distribution plot of mEPSC frequencies. All statistical analyses were performed two-sided. Bar graphs show mean + s.e.m. Individual data points are neurons. Source data are provided as a Source Data file.

Excitability parameters	1US recent							1US remote						
	tdTom-			tdTom+			p value	tdTom-			tdTom+			p value
**Membrane capacitance (pF)**	114.32	±	7.12	115.01	±	5.09	0.94	98.78	±	7.22	111.62	±	6.38	0.20
**RMP (mV)**	-64.92	±	0.83	-64.84	±	0.91	0.95	-66.33	±	1.47	-66.68	±	0.72	0.84
**Input resistance (MΩ)**	112.36	±	12.16	105.41	±	10.03	0.66	125.65	±	13.88	128.24	±	10.48	0.88
**τ_m (ms)**	22.33	±	3.29	20.55	±	2.04	0.87	22.77	±	2.41	25.14	±	2.05	0.46
**Sag ratio (%)**	13.35	±	1.89	11.48	±	1.42	0.44	9.05	±	1.96	7.14	±	1.72	0.49
**Current threshold (rheobase) (pA)**	134.12	±	10.54	141.18	±	11.75	0.66	129.23	±	14.43	125.00	±	10.78	0.82
**Ratio ISI_1/ISI_n**	0.60	±	0.06	0.67	±	0.05	0.38	0.52	±	0.06	0.56	±	0.04	0.58
**50% AP width (ms)**	0.73	±	0.02	0.72	±	0.02	0.94	0.70	±	0.02	0.71	±	0.02	0.79
**dv/dt_{max} (mV/ms)**	457.65	±	19.56	446.92	±	18.97	0.70	476.52	±	25.17	476.97	±	20.64	0.99
**dv/dt_{min} (mV/ms)**	-100.98	±	3.30	-99.53	±	3.11	0.75	-105.23	±	4.38	-103.19	±	3.44	0.72
**Voltage (firing) threshold (mV)**	-38.61	±	0.78	-37.75	±	0.84	0.66	-38.99	±	0.64	-39.48	±	0.78	0.63
**fAHP (mV)**	-6.03	±	0.41	-7.18	±	0.69	0.16	-5.38	±	0.52	-6.16	±	0.58	0.33
**mAHP (mV)**	-13.57	±	0.33	-14.17	±	0.59	0.38	-13.83	±	0.39	-14.71	±	0.44	0.15
**AP amplitude (mV)**	88.29	±	1.53	86.32	±	1.49	0.36	86.88	±	1.79	88.12	±	1.27	0.58

Supplementary Table 1. Excitability parameters of 1US CTC tagged PL PNs in comparison to non-tagged PNs at the recent and remote memory timepoint. Values are mean ± s.e.m. P-values were determined by two-sided Student *t* test or Mann Witney U-test depending on normality of the data distribution. RMP = resting membrane potential, τ_m = membrane time constant, AP = action potential, fAHP = fast after-hyperpolarization, mAHP = medium after-hyperpolarization, dv/dt_{max} = peak depolarization velocity, dv/dt_{min} = min peak repolarization velocity, ISI = interspike interval. All statistical analyses were performed two-sided.

Excitability parameters	3US recent							3US remote						
	tdTom-			tdTom+			p value	tdTom-			tdTom+			p value
**Membrane capacitance (pF)**	95.71	±	5.14	89.95	±	5.57	0.35	88.75	±	5.91	92.24	±	4.93	0.65
**RMP (mV)**	-68.64	±	1.80	-69.92	±	1.49	0.60	-67.52	±	0.91	-67.69	±	0.53	0.43
**Input resistance (MΩ)**	137.00	±	11.77	149.85	±	14.29	0.49	96.78	±	8.33	96.14	±	6.15	0.95
**τ_m (ms)**	30.18	±	4.14	27.78	±	3.57	0.66	16.75	±	1.28	19.79	±	1.83	0.31
**Sag ratio (%)**	13.29	±	1.44	10.50	±	1.96	0.26	16.49	±	2.31	16.84	±	1.48	0.90
**Current threshold (rheobase) (pA)**	129.64	±	8.13	136.00	±	13.37	0.69	128.21	±	8.59	151.07	±	14.56	0.33
**Ratio ISI_1/ISI_n**	0.52	±	0.03	0.52	±	0.04	0.95	0.54	±	0.05	0.56	±	0.03	0.67
**50% AP width (ms)**	1.10	±	0.04	1.15	±	0.03	0.25	1.06	±	0.03	1.05	±	0.03	0.81
**dv/dt_{max} (mV/ms)**	305.69	±	14.25	274.77	±	16.23	0.16	332.18	±	20.16	328.36	±	25.60	0.77
**dv/dt_{min} (mV/ms)**	-82.58	±	2.63	-77.89	±	2.22	0.18	-88.34	±	2.96	-88.38	±	2.78	0.99
**Voltage (firing) threshold (mV)**	-39.57	±	0.68	-37.95	±	0.68	0.10	-42.40	±	0.67	-41.45	±	0.58	0.29
**fAHP (mV)**	-4.83	±	0.55	-5.16	±	0.51	0.67	-3.91	±	0.46	-4.51	±	0.59	0.42
**mAHP (mV)**	-13.32	±	0.47	-13.92	±	0.57	0.42	-12.37	±	0.54	-13.01	±	0.44	0.37
**AP amplitude (mV)**	85.35	±	1.70	80.53	±	2.09	0.08	86.91	±	2.17	84.44	±	3.05	0.51

Supplementary Table 2. Excitability parameters of 3US CTC tagged PL PNs in comparison to non-tagged PNs at the recent and remote memory timepoint. Values are mean ± s.e.m. P-values were determined by two-sided Student *t* test or Mann Witney U-test depending on normality of the data distribution. RMP = resting membrane potential, τ_m = membrane time constant, AP = action potential, fAHP = fast after-hyperpolarization, mAHP = medium after-hyperpolarization, dv/dt_{max} = peak depolarization velocity, dv/dt_{min} = min peak repolarization velocity, ISI = interspike interval. All statistical analyses were performed two-sided.

Figure	Statistical test	Sample size
1c	One-way ANOVA $F_{2,9}$=43.69, p=0.000023, η^2 = 9.71;<br>Post-hoc Bonferroni tests: HC -4TM *vs*. HC +4TM, p=0.007, Cohen's d=4.22, 95% CI [0.457,2.648]; HC -4TM *vs*. CTC +4TM:, p=0.000019, Cohen's d=5.85, 95% CI [2.389, 4.581]; HC +4TM *vs*. CFC +4TM: p=0.002, Cohen's d=3.28, 95% CI [0.837,3.028]	HC- n=4<br>HC+4TM n=4<br>CFC+4TM n=4
1e	Unpaired t-test: t_{12}=2.8, p=0.015, Cohen's d=1.27, 95% CI [-29.35,-3.659]	mCherry n=9<br>hM4Di-mCherry n=10
1f	Unpaired t-test: t_{16}=0.6, p=0.563, Cohen's d=0.29, 95% CI [-7.804,13.9]	mCherry n=10<br>hM4Di-mCherry n=8
1i	Two-way RM ANOVA:<br>Population x Group: $F_{2,13}$=7.8, p=0.006<br>Population effect: $F_{1,13}$=171.6, p<0.000001<br>Group effect: $F_{2,13}$=5.5, p=0.019<br>Post-hoc Tukey tests: NS tdTom+ vs NS tdTom-, p=0.000079, Cohen's d=5.46, 95% CI [9.645,21.59]; 1US tdTom+ vs 1US tdTom-, p<0.000001, Cohen's d=6.24, 95% CI [23.16,35.10]; 3US tdTom+ vs 3US tdTom-, p=0.000023, Cohen's d=2.19, 95% CI [10.74,21.64]; NS tdTom+ vs 1US tdTom+, p=0.0004, Cohen's d=2.89, 95% CI [-24.16,-6.868]; NS tdTom+ vs 3US tdTom+, , p=0.88, Cohen's d=0.21, 95% CI [-9.866,6.689]; 1US tdTom+ vs 3US tdTom+, p=0.0008, Cohen's d=1.64, 95% CI [5.648,22.20]	NS n=5<br>1US n=5<br>3US n=6
2c	Resting membrane potential, Unpaired t-test: t_{32}=0.062, p=0.95, Cohen's d=0.09, 95% CI [-2.429, 2.589]<br>Input resistance, Unpaired t-test: t_{32}=0.4, p=0.66, Cohen's d=0.62, 95% CI [-39.03, 25.16]	tdTom- *N/n*=17/4<br>tdTom+ *N/n*=17/4
2d	Mixed-effects model:<br>Population x Current: $F_{24, 757}$=0.5, p=0.98;<br>Population: $F_{1,32}$=0.2, p=0.66	tdTom- *N/n*=17/4<br>tdTom+ *N/n*=17/4
2e	Resting membrane potential, Unpaired t-test: $t_{17.55}$=0.2, p=0.84, Cohen's d=0.30, 95% CI [-3.645,2.945]<br>Input resistance, Unpaired t-test: t_{25}=0.15, p=0.88, Cohen's d=0.21, 95% CI [-32.90,38.08]	tdTom- *N/n*=13/4<br>tdTom+ *N/n*=14/4
2f	Population x Current: $F_{24,595}$=0.2, p=0.99;<br>Population: $F_{1,25}$=0.01, p=0.92	tdTom- *N/n*=13/4<br>tdTom+ *N/n*=14/4
2g	Resting membrane, potential Unpaired t-test: $t_{20.9}$=1.5, p=0.16, Cohen's d=0.77, 95% CI [-6.048,3.488];<br>Input resistance, Unpaired t-test: t_{27}=0.7, p=0.50, Cohen's d=0.98, 95% CI [-25.44,51.14]	tdTom- *N/n*=14/3;<br>tdTom+ *N/n*=15/3
2h	Two-way RM ANOVA:<br>Population x Current: $F_{24, 648}$=0.3, p=0.999;<br>Population: $F_{1, 27}$=0.3, p=0.57	tdTom- *N/n*=14/3;<br>tdTom+ *N/n*=15/3
2i	Resting membrane potential, Mann-Whitney U-test: U=80.5, p=0.43;	tdTom- *N/n*=14/4;<br>tdTom+ *N/n*=14/4

	Input resistance, Unpaired t-test: t_{26}=0.062, *p*=0.95, Cohen's *d*=0.11, 95% CI [-17.87, 16.59]	
2j	Two-way RM ANOVA:<br>Population x Current: $F_{24, 624}$=0.97, *p*=0.50<br>Population effect $F_{1,26}$=2.498, *p*=0.126	tdTom- *N/n*=14/4;<br>tdTom+ *N/n*=14/4
3c	Mixed-effects model:<br>Population x Stimulation: $F_{7,214}$=0.2, *p*=0.98<br>Population: $F_{1,31}$=0.26, *p*=0.62	tdTom- *N/n*=15/7<br>tdTom+ *N/n*=18/7
3d	Unpaired t-test: t_{30}=1.2, *p*=0.23, Cohen's d=0.44, 95% CI [-0.166, 0.657];	tdTom- *N/n*=14/7<br>tdTom+ *N/n*=18/7
3e	Two-way RM ANOVA:<br>Population x ISI: $F_{1,27}$=0.6, *p*=0.42<br>Population: $F_{1,27}$ =0.1, *p*=0.77	tdTom- *N/n*=13/7<br>tdTom+ *N/n*=16/7
3f	Two-way RM ANOVA:<br>Population x Stimulation: $F_{1,2.4}$=2.5, *p*=0.078.<br>Population effect: $F_{1,4}$=5.3, *p*=0.027. Post-hoc Bonferroni test: 12µA tdTom+ vs. tdTom- *p*=0.028, Cohen's d=0.75, 95% CI [6.958,210.2]; 14µA tdTom+ vs. tdTom- *p*=0.007, Cohen's d=0.71, 95% CI [21.64,224.9]	tdTom- *N/n*=18/9<br>tdTom+ *N/n*=20/9
3g	Unpaired t-test: t_{34}=0.2, *p*=0.85, Cohen's d=0.91, 95% CI [-0.574,0.477]	tdTom- *N/n*=18/9<br>tdTom+ *N/n*=18/9
3h	Two-way RM ANOVA:<br>Population x ISI: $F_{1,38}$<0.001, p=0.99<br>Population $F_{1,38}$=5.1, *p*=0.031.	tdTom- *N/n*=20/9<br>tdTom+ *N/n*=20/9
3i	Mixed-effects model:<br>Population x Stimulation: $F_{7,188}$=0.6, *p*=0.76<br>Population $F_{1,27}$=1.0, *p*=0.33	tdTom- *N/n*=15/7<br>tdTom+ *N/n*=14/7
3j	Unpaired t-test: t_{20}=0.3, *p*=0.74, Cohen's d=0.14, 95% CI [-0.659,0.477]	tdTom- *N/n*=11/6<br>tdTom+ *N/n*=11/6
3k	Two-way RM ANOVA:<br>Population x ISI = $F_{1,22}$=0.6, *p*=0.42<br>Population $F_{1,22}$ = 1.3, *p* = 0.27	tdTom- *N/n*=12/6<br>tdTom+ *N/n*=12/6
3l	Mixed-effects model:<br>Population x Stimulation: $F_{7,241}$=2.5, *p*=0.016<br>Population effect $F_{1,35}$=4.6, *p*=0.038. Post-hoc Bonferroni test: 14 µA tdTom+ vs. tdTom- *p*=0.006, Cohen's d=0.70, 95% CI [33.12,316.3];	tdTom- *N/n*=17/8<br>tdTom+ *N/n*=20/8
3m	Unpaired t-test: t_{29}=0.91, *p*=0.37; Cohen's d=0.33, 95% CI [-0.685,0.263]	tdTom- *N/n*=15/7<br>tdTom+ *N/n*=16/7
3n	Two-way RM ANOVA:<br>Population x ISI $F_{1,27}$=0.3, *p*=0.533<br>Population $F_{1,27}$=6.7, *p*=0.016	tdTom- *N/n*=14/7<br>tdTom+ *N/n*=15/7
4c	Mixed-effects model:<br>Population x Segment: $F_{2,35}$=1.3, *p*=0.29<br>Population: $F_{1,29}$=0.6, *p*=0.45	Tuft: tdTom+, N/n=13/6, tdTom-, N/n=11/7

		Trunk: tdTom+, N/n=11/7, tdTom-, N/n=8/6<br>Basal: tdTom+, N/n=17/7, tdTom-, N/n=10/6
4d	Mixed-effects model:<br>Population x Segment: $F_{2,56}$=1.2, *p*=0.32<br>Population: $F_{1,30}$=6.4, *p*=0.017<br>Post-hoc Bonferroni test:<br>Tuft: *p*=0.27, Cohen's d=0.61, 95% CI [-0.3373,0.06198]<br>Trunk: *p*=0.031, Cohen's d=0.98, 95% CI [-0.4591,-0.01755]<br>Basal: *p*=0.59, Cohen's d=-0.47, 95% CI [-0.2914,0.09238]	Tuft: tdTom+, N/n=18/8, tdTom-, N/n=13/7<br>Trunk: tdTom+, N/n=18/8, tdTom-, N/n=12/7<br>Basal: tdTom+, N/n=17/8, tdTom-, N/n=14/7
4e	Mixed-effects model:<br>Population x Segment: $F_{2,31}$=0.5, *p*=0.90<br>Population: $F_{1,29}$=0.1, *p*=0.75	Tuft: tdTom+, N/n=9/5, tdTom-, N/n=7/4<br>Trunk: tdTom+, N/n=11/5, tdTom-, N/n=9/5<br>Basal: tdTom+, N/n=10/5, tdTom-, N/n=9/5
4f	Mixed-effects model:<br>Population x Segment: $F_{2,51}$=0.1, *p*=0.32<br>Population: $F_{1,29}$=4.7, *p*=0.039<br>Post-hoc Bonferroni test:<br>Tuft: *p*=0.25, Cohen's d=0.63, 95% CI [-0.2811,0.04881]<br>Trunk: *p>0.99,* Cohen's d=0.34, 95% CI [-0.2908,0.1407]<br>Basal: *p*=0.47, Cohen's d=0.62, 95% CI [-0.3136,0.08646]	Tuft: tdTom+, N/n=18/6, tdTom-, N/n=13/7<br>Trunk: tdTom+, N/n=18/6, tdTom-, N/n=13/7<br>Basal: tdTom+, N/n=15/5, tdTom-, N/n=9/7
5b left	Mann-Whitney U test: *U*=510, *p*=0.06	tdTom- *N/n*=37/15, tdTom+ *N/n*=37/15
5b right	Kolmogorov Smirnov test: *D*=0.05, *p*<0.000001	tdTom- N_{mEPSC}/n=3663/15, tdTom+ N_{mEPSC}/n=3663/15
5c left	Mann-Whitney U test: *U*=522, *p*=0.039	tdTom- *N/n*=37/15, tdTom+ *N/n*=37/15
5c right	Kolmogorov Smirnov test: *D*=0.06, *p*<0.001	tdTom- N_{mEPSC}/n=3662/15, tdTom+ N_{mEPSC}/n=3662/15
5e left	Mann-Whitney U test: *U*=199, *p*=0.46	tdTom- *N/n*=20/8, tdTom+ *N/n*=23/8
5e right	Kolmogorov Smirnov test: *D*=0.03, *p*=0.20	tdTom- N_{mEPSC}/n=1980/8, tdTom+ N_{mEPSC}/n=2277/8
5f left	Mann-Whitney U test: *U*=204, *p*=0.54	tdTom- *N/n*=20/8, tdTom+ *N/n*=23/8
5i right	Kolmogorov Smirnov test: *D*=0.03, *p*=0.24	tdTom- N_{mEPSC}/n=1980/8, tdTom+ N_{mEPSC}/n=2276/8
6c	Chi-square test predictor vs. random intercept model: $\chi^2(1)$ = 1.28, *p* = 0.26	tdTom-: N_{spines} = 2809, $n_{dendrite}$ = 41, n_{cell} = 13, $n_{section}$ = 12, tdTom+: N_{spines} = 5117,

		$n_{dendrite}$ = 63, n_{cell} = 19 , $n_{section}$ = 13
6d	Predictor model: b = -0.14, t = -2.05, p = 0.040	tdTom-: N_{spines} = 2809, $n_{dendrite}$ = 41, n_{cell} = 13, $n_{section}$ = 12, tdTom+: N_{spines} = 5117, $n_{dendrite}$ = 63, n_{cell} = 19 , $n_{section}$ = 13
6e	Chi-square test predictor vs. random intercept model: $\chi^2(1)$ = 0.10, p = 0.75	tdTom-: N_{spines} = 2809, $n_{dendrite}$ = 41, n_{cell} = 13, $n_{section}$ = 12, tdTom+: N_{spines} = 5117, $n_{dendrite}$ = 63, n_{cell} = 19 , $n_{section}$ = 13
6f	Predictor model: b = -0.12, t = -2.13, p = 0.033	tdTom-: N_{spines} = 4117, $n_{dendrite}$ = 61, n_{cell} = 15, $n_{section}$ = 13, tdTom+: N_{spines} = 4572, $n_{dendrite}$ = 60, n_{cell} = 15, $n_{section}$ = 13
6g	Predictor model: b = -0.19, t = -2.78, p = 0.006	tdTom-: N_{spines} = 4117, $n_{dendrite}$ = 61, n_{cell} = 15, $n_{section}$ = 13, tdTom+: N_{spines} = 4572, $n_{dendrite}$ = 60, n_{cell} = 15, $n_{section}$ = 13
6h	Chi-square test random intercept vs. fixed intercept model: $\chi^2(1)$ = 0.59, p = 0.44	tdTom-: N_{spines} = 4117, $n_{dendrite}$ = 61, n_{cell} = 15, $n_{section}$ = 13, tdTom+: N_{spines} = 4572, $n_{dendrite}$ = 60, n_{cell} = 15, $n_{section}$ = 13
6i	Predictor model: b = 0.25, t = 4.14, p < 0.001	tdTom-: $N_{dendrites}$ = 41, $n_{section}$ = 12, tdTom+: $N_{dendrites}$ = 62, $n_{section}$ = 13
6j	Chi-square test predictor vs. random intercept model: $\chi^2(1)$ = 3.50, p = 0.062<br>Predictor model: b = 0.26, t = 1.94, p = 0.053	tdTom-: $N_{dendrites}$ = 41, n_{cell} = 13, tdTom+: $N_{dendrites}$ = 62, n_{cell} = 19
6k	Predictor model: b = 0.46, t = 2.66, p = 0.008	tdTom-: $N_{dendrites}$ = 41, n_{cell} = 13, tdTom+: $N_{dendrites}$ = 62, n_{cell} = 19
6l	Chi-square test random intercept vs. fixed intercept model: $\chi^2(1)$ = 0.00, p = 1	tdTom-: $N_{dendrites}$ = 41, n_{cell} = 13, tdTom+: $N_{dendrites}$ = 62, n_{cell} = 19
6m	Chi-square test predictor vs. random intercept model: $\chi^2(1)$ = 0.92, p = 0.34	tdTom-: $N_{dendrites}$ = 61, n_{cell} = 15, tdTom+: $N_{dendrites}$ = 60, n_{cell} = 15

6n	Chi-square test predictor vs. random intercept model: $\chi^2(1) = 3.24$, p = 0.07<br>Predictor model: b = 0.15, t = 1.81, p = 0.07	tdTom-: $N_{dendrites}$ = 61, n_{animal} = *5*, tdTom+: $N_{dendrites}$ = 60, n_{animal} = 5
6o	Chi-square test predictor vs. random intercept model: $\chi^2(1) = 2.07$, p = 0.15	tdTom-: $N_{dendrites}$ = 61, n_{cell} = 15, tdTom+: $N_{dendrites}$ = 60, n_{cell} = 15
6p	Chi-square test random intercept vs. fixed intercept model: $\chi^2(1)$ = 0.00, p = 1	tdTom-: $N_{dendrites}$ = 61, n_{cell} = 15, tdTom+: $N_{dendrites}$ = 60, n_{cell} = 15

Supplementary Table 3. Extended data related to the statistical analyses presented in the figure panels of the main text. n = number of animals, N = number of neurons, unless otherwise indicated.
