# Supplementary material for: Threat intensity shapes cortical engram architecture supporting remote memory retrieval
Source: Nat Commun. 2026 Jun 11;17:7447. doi: 10.1038/s41467-026-74231-5 (PMC13408494; doi:10.1038/s41467-026-74231-5)
Supplement: Supplementary file 2 — Reporting Summary [file 41467_2026_74231_MOESM2_ESM.pdf]

nature portfolio

Corresponding author(s): Michel C. van den Oever; Priyanka Rao-Ruiz

Last updated by author(s): Jan 28, 2026

Reporting Summary

Nature Portfolio wishes to improve the reproducibility of the work that we publish. This form provides structure for consistency and transparency in reporting. For further information on Nature Portfolio policies, see our Editorial Policies and the Editorial Policy Checklist.

Statistics

For all statistical analyses, confirm that the following items are present in the figure legend, table legend, main text, or Methods section.

n/a	Confirmed	
☐	☒	The exact sample size (*n*) for each experimental group/condition, given as a discrete number and unit of measurement
☐	☒	A statement on whether measurements were taken from distinct samples or whether the same sample was measured repeatedly
☐	☒	The statistical test(s) used AND whether they are one- or two-sided<br>*Only common tests should be described solely by name; describe more complex techniques in the Methods section.*
☒	☐	A description of all covariates tested
☐	☒	A description of any assumptions or corrections, such as tests of normality and adjustment for multiple comparisons
☐	☒	A full description of the statistical parameters including central tendency (e.g. means) or other basic estimates (e.g. regression coefficient) AND variation (e.g. standard deviation) or associated estimates of uncertainty (e.g. confidence intervals)
☐	☒	For null hypothesis testing, the test statistic (e.g. *F*, *t*, *r*) with confidence intervals, effect sizes, degrees of freedom and *P* value noted<br>*Give P values as exact values whenever suitable.*
☒	☐	For Bayesian analysis, information on the choice of priors and Markov chain Monte Carlo settings
☐	☒	For hierarchical and complex designs, identification of the appropriate level for tests and full reporting of outcomes
☐	☒	Estimates of effect sizes (e.g. Cohen's *d*, Pearson's *r*), indicating how they were calculated

Our web collection on statistics for biologists contains articles on many of the points above.

Software and code

Policy information about availability of computer code

Data collection	Ethovision XT (Noldus), LSM510 (Zeiss), NIS-elements (Nikon), pClamp (Molecular Devices), IMARIS 9.9.1 (Oxford instruments)
Data analysis	Excel (Microsoft), Image J (NIH), MATLAB (Mathworks), Prism 9 (Graphpad), R (RStudios), MiniAnalysis (Synaptosoft); https://github.com/PantheaNemat/Mitric-Beerens_etal_2026_Nat.Commun.

For manuscripts utilizing custom algorithms or software that are central to the research but not yet described in published literature, software must be made available to editors and reviewers. We strongly encourage code deposition in a community repository (e.g. GitHub). See the Nature Portfolio guidelines for submitting code & software for further information.

Data

Policy information about availability of data

All manuscripts must include a data availability statement. This statement should provide the following information, where applicable:

- Accession codes, unique identifiers, or web links for publicly available datasets
- A description of any restrictions on data availability
- For clinical datasets or third party data, please ensure that the statement adheres to our policy

All data supporting the findings of this study are available within the paper and its Supplementary Information, including the Source data file.

Research involving human participants, their data, or biological material

Policy information about studies with human participants or human data. See also policy information about sex, gender (identity/presentation), and sexual orientation and race, ethnicity and racism.

Reporting on sex and gender	N/A
Reporting on race, ethnicity, or other socially relevant groupings	N/A
Population characteristics	N/A
Recruitment	N/A
Ethics oversight	N/A

Note that full information on the approval of the study protocol must also be provided in the manuscript.

Field-specific reporting

Please select the one below that is the best fit for your research. If you are not sure, read the appropriate sections before making your selection.

☒ Life sciences ☐ Behavioural & social sciences ☐ Ecological, evolutionary & environmental sciences

For a reference copy of the document with all sections, see nature.com/documents/nr-reporting-summary-flat.pdf

Life sciences study design

All studies must disclose on these points even when the disclosure is negative.

Sample size	Initial sample sizes were determined based on biological variance observed in our previous fear conditioning experiments and immunohistochemical quantifications (Matos et al., 2019, Nat Comm), electrophysiological recordings (Visser et al., 2022, Biol Psych) and spine analyses (Nemat et al., 2025, Neurbiol Learn Mem) to reach statistical significance with a power of at least 0.8. Some animals had to be excluded from the analyses (see below and Methods). Final sample sizes are indicated in the figure legends and supplementary table 3.
Data exclusions	Virus misplacements was a priori set as an exclusion criterion. In total 3 mice were excluded due to outlier analysis or virus misplacement. The exclusion of mice is described in more detail in the Methods (Statistical analysis).
Replication	Critical experiments with a statistically significant outcome were replicated successfully. Chemogenetic intervention effect on freezing (fig 1e-f) and engram reactivation (fig 1i) replicates previously reported data (Matos et al., 2019, Nat Comm). Significant electrophysiological findings (fig 3, 5) represent pooled data from independent batches that showed similar differences. The spine density measurements were replicated once in an independent batch and imaging method.
Randomization	For each experiment, mice were age- and weight-matched and randomly assigned to groups
Blinding	The investigators were not blinded for fear conditioning experiments, as freezing behavior was automatically scored using constant settings in Ethovision (Noldus). For electrophysiology, imaging and histological analyses, investigators were blinded to group allocation of individual animals.

Reporting for specific materials, systems and methods

We require information from authors about some types of materials, experimental systems and methods used in many studies. Here, indicate whether each material, system or method listed is relevant to your study. If you are not sure if a list item applies to your research, read the appropriate section before selecting a response.

Materials & experimental systems

n/a	Involved in the study	
☐	☒	Antibodies
☒	☐	Eukaryotic cell lines
☒	☐	Palaeontology and archaeology
☐	☒	Animals and other organisms
☒	☐	Clinical data
☒	☐	Dual use research of concern
☒	☐	Plants

Methods

n/a	Involved in the study	
☒	☐	ChIP-seq
☒	☐	Flow cytometry
☒	☐	MRI-based neuroimaging

Antibodies

Antibodies used	anti-RFP (1:2000, Tebu-Bio, Article Number 600-401-379); goat anti-rabbit, Alexa Fluor-633 (1:400, Molecular Probes, A21070); streptavidin conjugated to Alexa Fluor-488 (1:500, Molecular Probes, S-11223/S325); rat anti-Fos (1:1000, SySy, #226017); goat anti-rat Alexa Fluor-647 (1:400, Life technologies, A21247)
Validation	The antibodies are commercially available and successfully applied in previous studies (e.g. see Matos et al., Nat. Commun. 2019). Tissue stained without primary antibody served to rule out the possibility of non-specific binding of secondary antibody to the tissue. RFP: Statement of manufacturer - Assay by immunoelectrophoresis resulted in a single precipitin arc against anti-Rabbit Serum and purified and partially purified Red Fluorescent Protein (Discosoma). No reaction was observed against Human, Mouse or Rat serum proteins.

Animals and other research organisms

Policy information about studies involving animals; ARRIVE guidelines recommended for reporting animal research, and Sex and Gender in Research

Laboratory animals	Mice, TRAP2 x Ai14, age: 8-10 weeks old at the start of experiments. Fos2A-iCreER/+ (TRAP2) (stock #030323) and R26AI14/+ (AI14) (stock #007914) mouse lines were obtained from the Jackson Laboratory. TRAP2 mice were crossed with AI14 mice to acquire the double heterozygous (TRAP2;Ai14) used in this study.
Wild animals	N/A
Reporting on sex	Findings apply to male mice. Males were used in this study because animals had to be single-housed to avoid interaction of stress/re-establishement of group hierarchy with the fear conditioning paradigm. In addition, mice that underwent intracranial surgery had to be single-housed to prevent removal of clips and sutures. The Central Committee for Animal experiments (CCD) of The Netherlands and Animal Ethical care Committee of the Vrije Universiteit Amsterdam do not allow single-housing of female mice, excluding the possibility to use females.
Field-collected samples	N/A
Ethics oversight	The Central Committee for Animal experiments (CCD) of The Netherlands; Animal Ethical care Committee of the Vrije Universiteit Amsterdam (Instantie voor Dierenwelzijn)

Note that full information on the approval of the study protocol must also be provided in the manuscript.

Plants

Seed stocks	N/A
Novel plant genotypes	N/A
Authentication	N/A
