# Supplementary material for: Threat intensity shapes cortical engram architecture supporting remote memory retrieval
Source: Nat Commun. 2026 Jun 11;17:7447. doi: 10.1038/s41467-026-74231-5 (PMC13408494; doi:10.1038/s41467-026-74231-5)
Supplement: Supplementary file 3 — Transparent Peer Review file [file 41467_2026_74231_MOESM3_ESM.pdf]

nature portfolio

Peer Review File

Threat intensity shapes cortical engram architecture supporting remote memory retrieval

Corresponding Author: Dr Michel van den Oever

This file contains all reviewer reports in order by version, followed by all author rebuttals in order by version.

Version 0:

Reviewer comments:

Reviewer #1

(Remarks to the Author)
The current study seeks to reveal whether temporal changes in electrophysiological excitability and synaptic properties of PL pyramidal engram cells are modulated by the intensity of an aversive experience. Towards this goal, the authors use a combination of neural tagging, chemogenetics, electrophysiology, and structural spine analyses. In this way, the authors were able to reveal electrophysiological and structural plasticity signatures defining learning-activated neurons for both weaker and stronger threat memory. While the study is interesting, the level of mechanistic insight is somewhat lower than I would expect for a Nat Comms article. Moreover, I had some major concerns about the study itself that should be addressed:

1. The rationale that learning-activated neurons are engram neurons is incorrect. To be part of the engram, the cells that are activated during learning and must also be reactived during memory retrieval. This was something that was not tested in the study. Without those experiments, the text must be edited throughout to indicate that these are ensembles of learning-tagged PL neurons.

2. Why were analyses only restricted to layer 5 neurons? Tagging appears to occur across cortical layers (Fig 1b). Because of important laminar differences in cell physiology, connectivity, and morphology, it will be important to also report data for learning-tagged neurons in superficial layers.

3. Presumably, there are some neurons tagged in the strong memory ensemble that are fear memory encoding. Why does chemogenetic suppression (Fig 1f) of all of these neurons not affect the memory at all? One might expect it to be affected, but to a lower extent.

4. Additional cell counts for Fig 1 should be performed. How many cells are tagged for mild versus strong memory? How are these cells distributed across cortical layers? Moreover, are these tagged neurons reactivated at recent and remote time points (what % are reactivated, how are they distributed across layers, etc.)? These are important questions because results in Figure 1e,f could simply be due to more or less cells tagged in each group (reflecting a simple threshold effect). Or alternatively, it could potentially reflect a difference in the number of true engram cells that are incorporated into the learning-tagged neural ensemble.

5. Supplementary figure 4 is somewhat troubling. For animals with neurons that were tagged without the presentation of the US, the authors find significant differences in the cumulative probability plots for Tom+ vs Tom- neurons. This complicates interpretation of other Tom+ vs Tom- neuron analyses across their experimental groups. How many other differences are due to baseline differences in Tom+ versus Tom- neurons?

6. Lines 111-112 state “CTC-activated PL neurons are required for expression of remote mild, but not strong, threat memory” in reference to Fig 1e. However, a modest reduction in freezing from ~80% to ~60% in response to silencing the tagged ensemble does not imply that they are required for the memory. They more likely modulate it or are only a small part of the network.

7. For figure 2, were other measures like rheobase and firing threshold measured? These are also important metrics of excitability. If these are not altered, it is unclear what the alteration in AP discharge frequency could mean or how impactful this would be on in vivo activity and memory encoding. Indeed, no other metrics of intrinsic excitability were different across

groups.

8. Related to point 7, synaptic activity was not blocked during intrinsic excitability measurements. This greatly complicates interpretation of results as extrinsic factors like synaptic transmission can significantly impact excitability and obscure results.

9. Figure 3 results are somewhat confusing (described in lines 193-198). At the remote timepoint, on the one hand, electrically evoked EPSC amplitudes are reduced in TdTom+ neurons compared to TdTom- neurons. At the same time, there is increased presynaptic release probability onto TdTom+ vs TdTom- without a change in postsynaptic plasticity. This is difficult to reconcile and must be more fully discussed in the manuscript.

10. In Fig 4, the authors state on lines 265-268 that structural plasticity changes on oblique dendrites of 'engram' cells support mild threat memory. However, this was never tested. This is an observation, not necessarily causative. The language should be toned down.

Minor point
Line 29: I am not sure the correct word is maturation. Maybe plasticity or other word? Maturation, to me, implies development.

Reviewer #2

(Remarks to the Author)
In this manuscript the authors did a remarkable job of measuring in detail a large number of electrophysiological and histological parameters to find differences in these parameters between animals contextual threat conditioning associated with a one time and three time exposure of a footshock of 0.7 mA. Measurements were made in animals at two different time periods after conditioning namely 3-7 days and 28 -31 days. Comparisons were made between recruited and non-recruited neurons by working with a mouse strain that used an activity dependent marker which allowed expression of tdTomato to identify post-mortem, neurons in the PL that are presumably part of the engram. The functionality of these recruited neurons was assessed by an experiment in which the tagged neurons were also induced to express hM4Di by additional Cre-dependent aAV expressing hM4Di. Although the authors made an heroic effort to report all the different measurements made across all different neurons in different animals, it is a bit unfortunate that the results are not strikingly significantly different between different treatments.

The main question about this paper revolves about the results that come out of this functional testing experiment. Fig. 1e shows that only half of the mice that received CNO to inhibit the recruited neurons, showed decrease freezing levels (n=5), the other half maintains a baseline level. Thus, although the study makes an impressive contribution in mapping synaptic and structural properties of PL engram cells, the variability in behavioral response to CNO indicates the need to confirm whether all tagged cells are functionally involved in memory retrieval.

How to assess this?
First of all, a careful comparison should be made of the expression levels of hM4Di (or concomitant mCherry/tdTomato – it is, in fact, not completely clear to the reviewer whether these mice express both tdTomato and mCherry from the description of the experiment) between CNO responders and non-responders. How much variability is there is neuronal tagging between different animals? Maybe half of the animals do not express the tag, and concomitantly hm4Di.

Second, if both responders and non-responders both express similar levels of tdTomato/mCherry), then it may be that even if a neuron expresses hM4Di as a result of activity during training (CTC), does NOT imply that this neuron is also effectively recruited in the fear memory engram. To qualify as part of the fear memory engram, a recall sesssion should be included, before which tamoxifen is injected so that neurons that are activated during the recall will start to express hM4Di. Otherwise, it is not clear whether neurons expressing hM4Di are necessarily part of the memory engram. The authors do mention something of a memory retrieval session included in their behavioral paradigm, but it is unclear when this exactly occurs after the CTC training session.

Minor points:
1. Could the authors describe more clearly which animals were used across the different experiments? Notably, to what extent were the same animals used for behavioral experiments (Fig. 1, electrophysiological and imaging experiments?)
2. In supplementary Table 3, the authors describe that n=number of animals and N=number of neurons, unless otherwise indicated. However, for Fig. 5c, 5e & 5h and j, N = 3663, 3662, 1980 and 1980.

Reviewer #3

(Remarks to the Author)
Mitrić et al. investigate how the intensity of negatively valenced sensory stimuli influences the structural and functional properties of neuronal ensembles representing associative memory engrams in the prelimbic cortex (PL). Using TRAP2 knock-in mice, they performed electrophysiological and optical imaging analyses of PL neurons that were permanently labeled during contextual fear conditioning in a cFos- and drug-dependent manner with the genetically encoded fluorescent reporter tdTomato. The authors show that PL neurons activated by mild and strong unconditioned stimuli (1US and 3US,

respectively) exhibit distinct morphological and functional signatures. In the 1US condition, engram-allocated neurons display a progressive increase in total spine numbers due to the spatially restricted addition of new spines on oblique dendrites. In contrast, exposure to 3US results in a delayed dampening of intrinsic excitability. Nonetheless, both 1US and 3US elicited similar increases in presynaptic release probability and reductions in postsynaptic strength. Overall, this is a generally well-executed study that addresses an interesting and potentially clinically relevant topic. The paper is clearly written, the main results are convincing, and the statistical analyses are comprehensive. The manuscript could be a strong candidate for Nature Communications, provided that the following weaknesses are appropriately addressed.

Major Points
1) While the electrophysiological characterization of transiently activated neurons at two distinct time points (recent and remote) is elegant, some of the authors' conclusions appear premature or speculative. Specifically, it is conceivable that 1US and 3US activate different numbers and potentially different subtypes of excitatory neurons, which may have distinct intrinsic and synaptic properties. In Figure 1, the authors convincingly demonstrate that permanent labeling with tdTomato is both tamoxifen- and experience-dependent. They also show that CNO treatment reduces freezing in hM4Di mice after 1US, but not after 3US. However, the study does not directly test whether—and how—the ensembles activated by 1US and 3US differ from those engaged under home-cage conditions. Such differences could be both intrinsic and activity-dependent. At the very least, the authors should systematically analyze native Fos expression and recombination efficiency in TRAP2 mice across experimental conditions.
2) Along the same lines, the electrophysiological and synaptic properties of tdTomato-positive neurons should be compared side-by-side across 1US and 3US groups—not just versus randomly selected unlabeled neurons. This would strengthen claims regarding stimulus-specific adaptations.
3) The conclusion that mild stimulation increases structural connectivity is based solely on spine imaging. However, due to the relatively low resolution of optical imaging (especially at 40x with digital zoom), spine counts may be underestimated and spine classification may be inaccurate. More importantly, no evidence is provided to confirm that the newly formed spines are innervated, let alone the identity or origin of presynaptic axons. mEPSC frequency increases are not necessarily indicative of new functional synapses, especially in light of reported increases in presynaptic release probability.

Other Points
4) The behavioral paradigm used appears to follow standard contextual fear conditioning (CFC). Thus, referring to it as "contextual threat conditioning" may be misleading. Also, the manuscript should consistently use the standard abbreviation "4-OHT" for 4-hydroxytamoxifen.
5) The authors discuss how their findings are supported by prior associative fear engram studies. However, most of those studies used strong conditioning protocols. In particular, Matos et al., 2019 (from one of the corresponding authors) showed that chemogenetic silencing of mPFC engram cells 30 days after 3US reduced freezing, contrary to what is shown in Figure 1f. This discrepancy should be acknowledged and discussed.
6) It is surprising that a single shock delivered 28 days prior leads to ~80% freezing in most mice (Fig. 1). Are there prior reports showing such robust long-term memory after a single shock? The similarity in freezing between 1US and 3US groups should be contextualized.
7) Fig. 1b: The staining and image quality are suboptimal and may need improvement for publication.
8) Fig. 2: Statistical comparisons should be indicated directly on the graphs, as p-values are not shown in the figure.
9) Fig. 2a: Pooling recent timepoints (3–7 days) could obscure day-specific variability in excitability, as prior studies in other memory circuits have shown changes across this window.
10) As mentioned earlier, the current imaging configuration (40x with digital zoom) may lack the resolution necessary for accurate spine classification. Reanalysis using higher magnification optics—or ideally, electron microscopy—would greatly strengthen the conclusions about structural remodeling.
11) Introduction, Line 51: The statement that memory retrieval leads to partial reactivation of engram cells should acknowledge that in the cortex, most neurons tagged during learning are not reactivated during retrieval (Roy et al, 2022).
12) Introduction, Line 64: There is an apparent contradiction between the idea that memory retrieval depends on engram reactivation and the notion that mPFC engrams are disengaged after strong conditioning. If lasting memories rely on engram cells for retrieval, how can they become dispensable after strong training? The logic here needs to be clarified.
13) Introduction, Lines 75–78: The use of vague terms like "adaptations" and "changes" could be replaced with more specific language to improve clarity.
14) Discussion, Lines 428–431: The statement that strong conditioning "disengages" PL engrams should be softened. While chemogenetic silencing data suggest a reduced contribution to remote retrieval, Fos expression still indicates initial engagement, and the persistent physiological changes observed may still influence broader circuit function.
15) Discussion, Line 438: The word should be "resilience," not "resiliance."
16) Discussion, Lines 490–494: The claim that increased spine density after 1US is consistent with interhemispheric PL connectivity reported by Lee et al. (2023) should be reconsidered. That study used 5 shocks (strong conditioning) and reported changes, which contradicts the current claim.
17) Several recent studies have shown that Hebbian mechanisms alone cannot explain engram allocation across distributed brain regions. The authors should discuss how their findings relate to this growing body of work.
18) Additionally, The authors should more thoroughly discuss learning-related changes in excitability and how their results align—or conflict—with earlier studies.

Version 1:

Reviewer comments:

Reviewer #1

(Remarks to the Author)
The authors have thoroughly addressed my concerns.

Reviewer #2

(Remarks to the Author)
My crucial point of criticism to their findings was the labeling of the neurons by exposure only to Ctx+US without recall to the Ctx. This was also raised and targeted in much more detail by reviewer 1 as main criticism to their work. It appears the authors have addressed this point by further experiments and reference to their previous work.

Reviewer #3

(Remarks to the Author)
The authors have adequately addressed all of my previous concerns. The revised manuscript is much improved, and I recommend it for publication. Congratulations on a very nice study! Anton Maximov.

Open Access This Peer Review File is licensed under a Creative Commons Attribution 4.0 International License, which permits use, sharing, adaptation, distribution and reproduction in any medium or format, as long as you give appropriate credit to the original author(s) and the source, provide a link to the Creative Commons license, and indicate if changes were made.
In cases where reviewers are anonymous, credit should be given to 'Anonymous Referee' and the source.
The images or other third party material in this Peer Review File are included in the article's Creative Commons license, unless indicated otherwise in a credit line to the material. If material is not included in the article's Creative Commons license and your intended use is not permitted by statutory regulation or exceeds the permitted use, you will need to obtain permission directly from the copyright holder.
To view a copy of this license, visit https://creativecommons.org/licenses/by/4.0/

NCOMMS-25-46802. Response to the reviewers

We would like to express our sincere gratitude to the editor and the reviewers for their positive comments and constructive remarks. Based on the reviewers' comments, we have performed a set of novel experiments and data analyses that together strengthen our findings and conclusions. For instance, the new data demonstrate differential reactivation of tagged PL neurons during retrieval of a mild versus strong threat memory and support our choice to study deep layer pyramidal engram cells. Moreover, we have made substantial revisions to the text to clarify methods and data interpretation, elaborate on discussion points, and acknowledge limitations of our study. Please find a point-by-point rebuttal (in green) to the reviewer's comments (in black) below.

REVIEWER COMMENTS

Reviewer #1 (Remarks to the Author):

The current study seeks to reveal whether temporal changes in electrophysiological excitability and synaptic properties of PL pyramidal engram cells are modulated by the intensity of an aversive experience. Towards this goal, the authors use a combination of neural tagging, chemogenetics, electrophysiology, and structural spine analyses. In this way, the authors were able to reveal electrophysiological and structural plasticity signatures defining learning-activated neurons for both weaker and stronger threat memory. While the study is interesting, the level of mechanistic insight is somewhat lower than I would expect for a Nat Comms article. Moreover, I had some major concerns about the study itself that should be addressed:

1. The rationale that learning-activated neurons are engram neurons is incorrect. To be part of the engram, the cells that are activated during learning and must also be reactived during memory retrieval. This was something that was not tested in the study. Without those experiments, the text must be edited throughout to indicate that these are ensembles of learning-tagged PL neurons.

We agree with the reviewer that engram cells are learning-activated neurons that should be (at least partially) reactivated during memory retrieval. In our previous study[1], on which the present data builds, we have already shown that learning-activated PL neurons (tagged using viral-TRAP) are preferentially reactivated during remote retrieval. We have performed a new experiment including no-shock (No US; i.e., context exposure alone), 1US and 3US CTC groups (Fig. 1g-, Suppl. Fig. 2). This confirms that preferential reactivation of tagged PL neurons occurs in TRAP2-tdTom mice, similar to what has been observed by others[2, 3]. Notably, Fos is expressed in tagged neurons above chance levels across experimental groups (Supplementary Fig. 2b), however, 1US CTC-tagged PL neurons show enhanced reactivation compared with reactivation rates in No US- and 3US-tagged neurons.

Line 116-125:
Next, endogenous reactivation of tagged PL neurons during memory retrieval was examined after 1US and 3US CTC, as well as in a No US (no shock control) group (Fig. 1g). Colocalization analysis of Fos (induced during the retrieval test) with tdTom$^+$ and tdTom$^-$ neurons revealed that tagged PL neurons were preferentially reactivated across all groups, with 1US CTC mice showing the highest reactivation level (Fig. 1h-I, Supplementary Figure 2a-e). This is in line with the threat intensity-dependent effect of chemogenetic suppression of tagged PL neurons on remote memory expression. Interestingly, the reactivation rate was similar in the No US and 3US CTC groups, suggesting this level may reflect recognition of the context alone.

2. Why were analyses only restricted to layer 5 neurons? Tagging appears to occur across cortical layers (Fig 1b). Because of important laminar differences in cell physiology, connectivity, and morphology, it will be important to also report data for learning-tagged neurons in superficial layers.

The reviewer raises a relevant question. Supported by literature and novel data, we now provide a better explanation for studying layer 5 (L5) pyramidal neurons. Firstly, our choice was initially based on the original study by DeNardo et al. (2019) using TRAP2 mice that were crossed with the Ai14 (tdTomato reporter) line (same as our mice). They found that the fraction of PL neurons that were reactivated during retrieval of remote fear memory was larger in the deep layers (L5/6) compared with superficial layers (L1-3). A more recent study further confirmed the necessity of specifically deep-layer PL engram cells in expression of remote fear memory[3].
Secondly, we choose to examine L5 pyramidal neurons because they have a longer apical dendrite that spans multiple layers, enabling spine analyses of trunk dendrites in L2/3 and tuft dendrites in L1/2.
Thirdly, we now trained a new group of TRAP2-tdTom mice to quantify the distribution of tagged and reactivated neurons across superficial (L2/3) and deep (L5/6) layers after 1US and 3US CTC, and compared this with mice that did not receive a foot-shock in the conditioning context (No US). In the new Suppl. Fig 2f-g, we show the fraction of tagged (tdTom$^+$) neurons in L2/3 or L5/6 as the subset of the total tagged population in L2-6, and the fraction of reactivated neurons (tdTom$^+$Fos$^+$) in L2/3 or L5/6 as the subset of all reactivated neurons in L2-6, similar to DeNardo et al., 2019[2]. This new data demonstrates that most of the tagged and reactivated neurons are indeed located in the deep layers. Note that for L5/6, the reactivation fraction of 1US tagged neurons might seem lower than in No US or 3US (Suppl. Fig 2g), but we did not detect a significant interaction effect in the data. ANOVA revealed a main effect of layer only and this was supported by post hoc analyses. Please also note that the total reactivation rate is higher after 1US compared to No US and 3US (see Fig. 1i).
In the results section, we describe these arguments to support our choice and claims made for L5 PL engram cells:

Line 137-143:
Whole-cell electrophysiological recordings were performed from layer 5 pyramidal neurons (PNs) given their prevalent cortical and subcortical connections[4], specific

relevance in remote memory expression[3], and because they have an extensive apical dendrite relevant for structural analyses of distinct dendritic segments. Moreover, of all PL engram cells, the largest fraction is located in the deep cortical layers and reactivated during remote memory retrieval (Supplementary Fig. 2f-g)[2].

We acknowledge that laminar differences in pyramidal cell properties exist and cannot exclude that L2/3 pyramidal engram cells may also undergo learning-induced changes. However, to obtain the same extensive dataset for pyramidal neurons in superficial layers would require several years of additional work. Therefore, we think a comparison of physiological and structural analyses of neurons in superficial and deep after different threat intensities is beyond the scope of the current study. We describe this limitation now in the discussion section.

Line 408-412:
However, our study has some limitations. Firstly, we specifically focussed on layer 5 PNs. Given that PNs in superficial (layer 2/3) and deep (layer 5/6) layers differ in physiological and morphological properties, as well as their connectivity[5], it is possible that CTC induces changes in layer 2/3 PNs that are distinct from those in layer 5.

Importantly, our claims made for L5 pyramidal cells remain valid for these specific engram cells. For clarity, we added ‘layer 5 pyramidal’ to ‘engram cells’ at several locations in the main text.

3. Presumably, there are some neurons tagged in the strong memory ensemble that are fear memory encoding. Why does chemogenetic suppression (Fig 1f) of all of these neurons not affect the memory at all? One might expect it to be affected, but to a lower extent.

This is indeed an intriguing question. We would like to emphasize that we previously found that chemogenetic suppression of 3US CFC-tagged PL neurons has no effect on remote memory expression[1], which we now replicated in TRAP2 mice.
As shown in Fig. 1i, the reactivation rate of tdTom+ neurons after retrieval of 3US memory is significantly lower than the reactivation rate during retrieval of 1US memory. Importantly, 3US reactivation does not differ from the reactivation rate in the No US group. Therefore, the 3US reactivation rate might primarily reflect memory of the context, and not the fear association. We now describe this possibility in the results:

Line 123:
Interestingly, the reactivation rate was similar in the No US and 3US CTC groups, suggesting this level may reflect recognition of the context alone.

Our findings are in line with data by DeNardo et al. (2019), who also did not detect a difference in PL engram reactivation rate between cells tagged during strong fear conditioning (8 US) and a no-shock group[2]. Based on the electrophysiological and structural analyses, the reduced excitability and absence of an increase in spine density

in PL neurons tagged during 3US CTC likely underlies their reduced reactivation rate during remote memory expression.

4. Additional cell counts for Fig 1 should be performed. How many cells are tagged for mild versus strong memory? How are these cells distributed across cortical layers? Moreover, are these tagged neurons reactivated at recent and remote time points (what % are reactivated, how are they distributed across layers, etc.)? These are important questions because results in Figure 1e,f could simply be due to more or less cells tagged in each group (reflecting a simple threshold effect). Or alternatively, it could potentially reflect a difference in the number of true engram cells that are incorporated into the learning-tagged neural ensemble.

We thank the reviewer for this insightful suggestion. In the new independent cohort of TRAP2-tdTom mice, we now performed additional cell counts to determine how many cells are tagged after mild vs. strong threat conditioning, and how these are distributed and reactivated across cortical layers (Suppl. Fig 2). We found no difference in the percentage and distribution of tagged neurons between 1US and 3US across superficial (L2/3) and deep layers (L5/6). Both 1US and 3US tagged neurons were preferentially reactivated, although in the 3US group to a lower extent. We have not studied reactivation during recent memory retrieval, as we and others previously found that the PL engram is not necessary for retrieval of recent memory[1, 6, 7]. Importantly, this shows the difference in effect of chemogenetic inhibition on freezing behavior was not due to differences in tagging after 1US and 3US CTC.

Nonetheless, the reviewer raises a good point about ‘true engram cells that are incorporated into the learning-tagged neural ensemble’. Since activity of 3US-tagged neurons is not required for remote memory retrieval and these cells are reactivated to the same level as No US controls, these neurons are likely not true engram neurons based on the criterium of ecphory, i.e., an engram is expressed behaviorally through interactions with retrieval cues[8]. Therefore, we refer to 3US CFC-tagged neurons in the main text as ‘tagged’ or ‘learning-activated’ neurons instead of engram neurons.

5. Supplementary figure 4 is somewhat troubling. For animals with neurons that were tagged without the presentation of the US, the authors find significant differences in the cumulative probability plots for Tom+ vs Tom- neurons. This complicates interpretation of other Tom+ vs Tom- neuron analyses across their experimental groups. How many other differences are due to baseline differences in Tom+ versus Tom- neurons?

We think this comment reflects a lack of clarity in the explanation we provided for the interpretation of the cumulative distribution plots. We now provide a better explanation based on the following arguments:
Firstly, we think a significant difference in the cumulative distribution plots is only meaningful when there is also a significant difference between the respective means of the amplitude or frequency (shown in the bar graphs of Suppl. Fig 5). In the statistical analysis of cumulative distribution plots, the amplitude/frequency of every mEPSC is

included as an individual data point and combined for all recorded cells within a group, thereby substantially increasing the number of observations. This can inflate small differences, as evident from the negligible, but significant, difference in the distribution plots of Suppl. Fig 5. We mainly included these plots to demonstrate how the curve of the amplitude/frequency of tagged neurons shifted compared non-tagged neurons. To avoid confusion about the interpretation of these plots, we now describe that the distribution curves were only compared when the difference between the means reached significance. Therefore, we removed statistical analyses of these plots when the means did not reach significance.

Line 225-228:
First, the means of mEPSC amplitudes or frequencies were compared and only when this reached significance, cumulative distribution plots were compared to demonstrate a potential shift in the curves.

Secondly, tdTom+ neurons in the No US group may not reflect a baseline state of PL neurons. Although the No US group did not receive a foot-shock, these mice were exposed to a novel context and they likely form a neutral contextual memory. This is supported by the new observation that tagged PL neurons in the No US group are preferentially reactivated when the mice are re-exposed to this context (Fig. 1i and Suppl. Fig. 2). Please note that apart from the cumulative distribution plots, we did not find other differences between tdTom+ and tdTom- neurons in the No US group, indicating that the differences that we find in the 1US and 3US groups specifically develop after an aversive experience.

6. Lines 111-112 state “CTC-activated PL neurons are required for expression of remote mild, but not strong, threat memory” in reference to Fig 1e. However, a modest reduction in freezing from ~80% to ~60% in response to silencing the tagged ensemble does not imply that they are required for the memory. They more likely modulate it or are only a small part of the network.

We agree with the reviewer’s suggestion that CTC-activated PL neurons are likely only a small part of the network. However, the significant reduction in freezing levels after chemogenetic suppression of these specific neurons does indicate that they functionally contribute to the engram network during expression of a mild threat memory. Please note that a partial reduction in freezing upon silencing of PL engram cells is a common observation[1, 3, 6]. Memories are represented in connected engrams across multiple brain regions and artificial reactivation of engrams across multiple brain regions is required to resemble the level of natural recall[9]. Therefore, inhibition of an engram in a single brain region will likely not result in a complete inability to retrieve/express the memory. We softened the statement in the results section:

Line 127:
…CTC-activated PL neurons mediate expression of mild, but not strong, remote threat memory.

We would also like to refer to the potential explanation related to this comment in the discussion section:

Lines 295-298:
This intervention results in a partial reduction of freezing behaviour, likely because the PL functions as a hub in a larger engram circuit. Suppression of engram cells in a distributed network involving multiple brain regions, e.g., including the basolateral amygdala[6], may be required to fully block memory expression[9].

7. For figure 2, were other measures like rheobase and firing threshold measured? These are also important metrics of excitability. If these are not altered, it is unclear what the alteration in AP discharge frequency could mean or how impactful this would be on in vivo activity and memory encoding. Indeed, no other metrics of intrinsic excitability were different across groups.

Rheobase and firing threshold were both measured and present in supplementary tables 1 and 2 as 'current threshold' and 'voltage threshold', respectively. These measures were not different between tdTom+ and tdTom- neurons in both 1US and 3US conditions. To avoid confusion, we renamed these parameters to 'current threshold (rheobase)' and 'voltage (firing) threshold' in Suppl. Tables 1 and 2.

Although these and other metrics of intrinsic excitability were indeed not different, the alteration in AP discharge frequency can nonetheless reflect a reduction in excitability, likely driven by an extrinsic factor (see rebuttal at point 8, below). Regarding the impact of changes after 3US CTC, we hypothesize that the reduced excitability of tdTom+ neurons after strong CTC, combined with reduced eEPSC amplitude and spine size, reduces the probability that 3US tagged PL neurons are reactivated during memory retrieval. The latter is supported by the new reactivation analysis presented in Fig. 1g-i. We have included this in the discussion.

Lines 339-341:
Thus, decreased excitability, alongside reduced postsynaptic strength and spine size on oblique dendrites, potentially decreases the probability that strong threat-activated PL neurons become reactivated during remote memory retrieval. This is further supported by our finding that the reactivation level of these neurons during remote memory retrieval is lower than the reactivation rate of mild threat-activated PL neurons.

8. Related to point 7, synaptic activity was not blocked during intrinsic excitability measurements. This greatly complicates interpretation of results as extrinsic factors like synaptic transmission can significantly impact excitability and obscure results.

It is not uncommon to record excitability parameters without blocking synaptic activity in the slice preparation[10, 11, 12, 13, 14], and that it was also not our aim to specifically isolate intrinsic parameters that affect excitability. Our approach has the advantage to detect the contribution of both intrinsic, as well as extrinsic, factors to excitability changes. For this

reason, we also do not claim that 'intrinsic excitability' of tagged neurons is affected after 3US CTC, but we refer to 'excitability' only. As recorded intrinsic excitability parameters were not changed, extrinsic factors, like inhibitory drive, most likely altered the excitability of 3US-tagged neurons. Please note that we acknowledge that we did not block synaptic transmission during these recordings in the discussion section, and that we speculate about extrinsic factors that could mediate the change in excitability after strong CTC:

Line 341-350:
The decrease in AP discharge frequency after strong CTC is likely not driven by a cell intrinsic mechanism, as passive membrane and AP properties of tagged PL neurons were unaltered. Instead, it may be mediated by differences in the weight of excitatory or inhibitory inputs, as synaptic transmission was not blocked during these recordings. Although the eEPSC amplitude is reduced in PL engram cells after both mild and strong CTC, excitability is only affected after strong CTC, suggesting that attenuated excitatory drive alone does not account for the reduced excitability. Our analyses do not rule out that enhanced inhibitory input onto strong CTC-activated PL neurons underlies their reduced excitability, which remains to be elucidated.

9. Figure 3 results are somewhat confusing (described in lines 193-198). At the remote timepoint, on the one hand, electrically evoked EPSC amplitudes are reduced in TdTom+ neurons compared to TdTom- neurons. At the same time, there is increased presynaptic release probability onto TdTom+ vs TdTom- without a change in postsynaptic plasticity. This is difficult to reconcile and must be more fully discussed in the manuscript.

Although a decrease in presynaptic release probability is generally associated with reduced evoked EPSCs, a reduction in postsynaptic strength can occur alongside increased presynaptic release probability[15]. It may reflect forms of homeostatic synaptic plasticity. Increased presynaptic release probability may potentiate learning-activated and/or new synapses on tagged neurons, while the reduction in postsynaptic strength occurs at non-activated synapses to maintain overall synaptic weight of the cell. Alternatively, the reduction in postsynaptic strength (through reduced eEPSC amplitude and smaller spines) may be compensated by an increase in the probability of presynaptic vesicle fusion to maintain synaptic weigth[16, 17].

For clarity, we have adapted the discussion section related to this point:

Line 384-391:
Alternatively, the increase in presynaptic release probability may result from homeostatic synaptic plasticity[16], such that the reduction in postsynaptic strength (through decreased eEPSC amplitude and smaller spines) may be compensated by an increase in the probability of presynaptic vesicle release to maintain synaptic weigth[17]. Whether the increased presynaptic release probability serves to strengthen connectivity of PL engrams cells with one or more input regions in a threat intensity-dependent manner, or to compensate for the reduced postsynaptic strength, remains an important topic for future research.

10. In Fig 4, the authors state on lines 265-268 that structural plasticity changes on oblique dendrites of 'engram' cells support mild threat memory. However, this was never tested. This is an observation, not necessarily causative. The language should be toned down.

We agree with the reviewer that we do not show a causal relationship between structural plasticity and mild threat memory retrieval. This is not what we intended to state. We meant that structural plasticity takes place only on those neurons that are also functionally involved in memory retrieval, thus those activated during mild, but not strong CTC. We have rephrased the sentence for clarity:

Line 213-217:
These findings reveal that compared with non-tagged PNs, structural plasticity through increased spine density develops progressively and exclusively on oblique dendrites of tagged PNs after mild CTC, corresponding with their selective functional involvement in retrieval of the mild remote threat memory.

Minor point
Line 29: I am not sure the correct word is maturation. Maybe plasticity or other word? Maturation, to me, implies development.

We have replaced the word 'maturation' by:
- Abstract (line 31): physiological and structural properties
- Discussion (line 317): synaptic adaptations
- Discussion (line 430): circuits

Or removed it without replacement:
- Discussion (line 329): …to support their gradual ~~maturation and~~ functional integration…

Reviewer #2 (Remarks to the Author):

In this manuscript the authors did a remarkable job of measuring in detail a large number of electrophysiological and histological parameters to find differences in these parameters between animals contextual threat conditioning associated with a one time and three time exposure of a footshock of 0.7 mA. Measurements were made in animals at two different time periods after conditioning namely 3-7 days and 28 -31 days. Comparisons were made between recruited and non-recruited neurons by working with a mouse strain that used an activity dependent marker which allowed expression of tdTomato to identify post-mortem, neurons in the PL that are presumably part of the engram. The functionality of these recruited neurons was assessed by an experiment in which the tagged neurons were also induced to express hM4Di by additional Cre-dependent aAV expressing hM4Di. Although the authors made an heroic effort to report all the different measurements made

across all different neurons in different animals, it is a bit unfortunate that the results are not strikingly significantly different between different treatments.

The main question about this paper revolves about the results that come out of this functional testing experiment. Fig. 1e shows that only half of the mice that received CNO to inhibit the recruited neurons, showed decrease freezing levels (n=5), the other half maintains a baseline level. Thus, although the study makes an impressive contribution in mapping synaptic and structural properties of PL engram cells, the variability in behavioral response to CNO indicates the need to confirm whether all tagged cells are functionally involved in memory retrieval.

How to assess this?
First of all, a careful comparison should be made of the expression levels of hM4Di (or concomitant mCherry/tdTomato – it is, in fact, not completely clear to the reviewer whether these mice express both tdTomato and mCherry from the description of the experiment) between CNO responders and non-responders. How much variability is there is neuronal tagging between different animals? Maybe half of the animals do not express the tag, and concomitantly hm4Di.

We thank the reviewer for raising this relevant point. Please note that we have previously reported a difference in the functional involvement of 1US and 3US CTC-tagged PL neurons in remote memory expression using a viral-TRAP approach[1], and consider it of importance that we now replicated this effect in TRAP2-tdTom mice.

For the experiment in Fig. 1d-f, TRAP2-tdTom mice expressed hM4Di-mCherry (or mCherry alone in control mice), resulting in expression of tdTom and mCherry in learning-activated PL neurons. To determine whether tagging differences affected the freezing levels in these animals, we quantified the tdTom/mCherry-tagged neurons in the 1US CTC hM4Di group from figure 1e, and then divided the animals into the low (< 60%, n=5) and high (> 60%, n=5) freezing subgroups the reviewer refers to (Rebuttal Figure 1, below). This does not reveal a difference in the density of tagged PL neurons between high and low freezers, indicating that this subdivision is not caused by a difference in tagging.

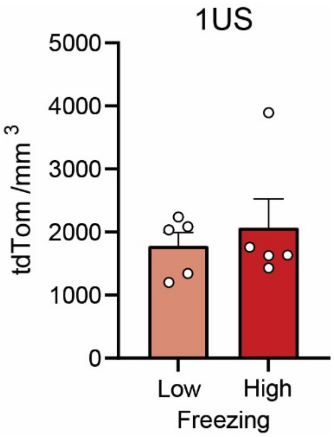

***Rebuttal Figure 1**. Engram tagging levels do not explain the difference in freezing levels within the 1US CTC hM4Di group. Mann-Whitney test: U=12, p > 0.999.*

Second, if both responders and non-responders both express similar levels of tdTomato/mCherry), then it may be that even if a neuron expresses hM4Di as a result of activity during training (CTC), does NOT imply that this neuron is also effectively recruited in the fear memory engram. To qualify as part of the fear memory engram, a recall sesssion should be included, before which tamoxifen is injected so that neurons that are activated during the recall will start to express hM4Di. Otherwise, it is not clear whether neurons expressing hM4Di are necessarily part of the memory engram. The authors do mention something of a memory retrieval session included in their behavioral paradigm, but it is unclear when this exactly occurs after the CTC training session.

We agree with the reviewer that when a neuron becomes activated during training, it does not automatically imply that this neuron is also effectively recruited into the memory engram. In fact, this is also what we show when mice undergo strong CTC, as those neurons are not functionally required to express the remote threat memory. However, we respectfully disagree that we should tag neurons during a recall session to qualify them as being part of the engram for the following reasons:

Firstly, we provide causal evidence for the functional contribution of 1US training-activated PL neurons to memory expression in Fig 1d-f, replicating our previous finding[1]. In this experiment, mice underwent a retrieval session 28 days after conditioning (see Fig.1d). During this session, the tagged neurons were chemogenetically inhibited. We have revised the text in the results to clarify this:

Line 107-110:
To assess the functional role of 1US and 3US CTC-tagged PL neurons, mice received Cre-dependent AAV expressing hM4Di-mCherry (or mCherry alone) in the PL to enable chemogenetic inhibition of tagged neurons during remote memory retrieval.

Secondly, in a new independent group of TRAP2-tdTom mice, we show that learning-activated PL neurons are also preferentially reactivated during remote memory retrieval (Fig. 1g-i and Suppl. Fig 2). We acknowledge that after 1US CTC, not all learning-activated neurons are engram cells, as not all tagged neurons are reactivated upon

memory retrieval. However, the same argument applies to the PL population that is activated during a first retrieval test, as also only a subset of those neurons is reactivated during a second retrieval test[2]. The non-reactivated fraction of a tagged population may reflect non-engram cells, but it is also possible that memory retrieval may require reactivation of only a subset of the total engram population. The composition of this subset may differ with each retrieval event.

Thirdly, according to the engram definition, an engram is a persistent change in the brain resulting from a specific experience or event [8, 18]. To identify experience-induced changes that support memory expression the first time it is retrieved, it is necessary to tag neurons activated during training and to study the changes induced by training in the absence of a retrieval session. Therefore, we omitted the retrieval session for all electrophysiological and structural analyses. This is an established approach to study engrams[7, 19, 20, 21].

Minor points:
1. Could the authors describe more clearly which animals were used across the different experiments? Notably, to what extent were the same animals used for behavioral experiments (Fig. 1, electrophysiological and imaging experiments?)

To clarify this, we added the following text to the Methods section:

Line 440-444:
Independent cohorts were used for experiments in Fig. 1 (and distinct groups for panels a-c, d-f and g-i), as well as the electrophysiological experiments in Figs 2, 3 and 5. Spine density quantifications (Fig. 4) were performed on neurons that were filled with biocytin during eEPSC recordings (Fig. 3). Spine reconstruction and classification (Fig. 6) were performed on neurons that were filled with biocytin during mEPSC recordings (Fig. 5).

N.B. the latter information is described in the methods section (line 618-619 and 628-629) and in the results section (line 196-200 and 246-248).

2. In supplementary Table 3, the authors describe that n=number of animals and N=number of neurons, unless otherwise indicated. However, for Fig. 5c, 5e & 5h and j, N = 3663, 3662, 1980 and 1980.

We thank the reviewer for noticing this error. In those figures, N represents the number of mEPSCs detected. We have corrected this.

Reviewer #3 (Remarks to the Author):

Mitrić et al. investigate how the intensity of negatively valenced sensory stimuli influences the structural and functional properties of neuronal ensembles representing associative memory engrams in the prelimbic cortex (PL). Using TRAP2 knock-in mice, they

performed electrophysiological and optical imaging analyses of PL neurons that were permanently labeled during contextual fear conditioning in a cFos- and drug-dependent manner with the genetically encoded fluorescent reporter tdTomato. The authors show that PL neurons activated by mild and strong unconditioned stimuli (1US and 3US, respectively) exhibit distinct morphological and functional signatures. In the 1US condition, engram-allocated neurons display a progressive increase in total spine numbers due to the spatially restricted addition of new spines on oblique dendrites. In contrast, exposure to 3US results in a delayed dampening of intrinsic excitability. Nonetheless, both 1US and 3US elicited similar increases in presynaptic release probability and reductions in postsynaptic strength. Overall, this is a generally well-executed study that addresses an interesting and potentially clinically relevant topic. The paper is clearly written, the main results are convincing, and the statistical analyses are comprehensive. The manuscript could be a strong candidate for Nature Communications, provided that the following weaknesses are appropriately addressed.

Major Points

1) While the electrophysiological characterization of transiently activated neurons at two distinct time points (recent and remote) is elegant, some of the authors' conclusions appear premature or speculative. Specifically, it is conceivable that 1US and 3US activate different numbers and potentially different subtypes of excitatory neurons, which may have distinct intrinsic and synaptic properties. In Figure 1, the authors convincingly demonstrate that permanent labeling with tdTomato is both tamoxifen- and experience-dependent. They also show that CNO treatment reduces freezing in hM4Di mice after 1US, but not after 3US. However, the study does not directly test whether—and how—the ensembles activated by 1US and 3US differ from those engaged under home-cage conditions. Such differences could be both intrinsic and activity-dependent. At the very least, the authors should systematically analyze native Fos expression and recombination efficiency in TRAP2 mice across experimental conditions.

We thank the reviewer for this relevant question. We have now performed a new experiment to quantify the number of tagged neurons in the PL after 1US and 3US CTC and compared this with a No US (no shock, context only) group (Suppl. Fig 2). Furthermore, we compared tagging in superficial layers 2/3 with deep layers 5/6. We did not find differences in the percentage of tagged neurons between conditions, and this did also not differ between layers. Hence, this data does not point to intrinsic differences in the tagging between 1US and 3US CTC.

Although we did not determine which specific subtype of excitatory neuron we analyzed, we focused only on excitatory pyramidal neurons in layer 5 and performed within subject pairwise comparisons of tagged and neighboring non-tagged pyramidal cells in this layer to limit intrinsic variability between cells. Moreover, we did not observe differences in excitability and synaptic properties between tagged and non-tagged neurons in both 1US and 3US animals at the recent time point, suggesting that the tagged populations did not intrinsically differ and that changes observed at the remote timepoint developed as a result of the aversive experience. Furthermore, in addition to distinct changes, we found that 1US and 3US CTC-tagged pyramidal cells develop shared adaptations (reduced

eEPSC amplitude and spine head volume), which also suggests that these neurons are not intrinsically different.

For the native Fos analysis requested by the reviewer, we trained a new batch of TRAP2-tdTom mice. They either remained in their home-cage or were exposed to 1US CTC or 3US CTC (n = 4 per group). Mice were perfused 90 min after CTC to capture the peak of Fos expression. The percentage of Fos^+ cells differed significantly between groups (Rebuttal Figure 2, below). As evident from the graph, Fos expression is substantially lower in the HC group than the CTC groups, although this difference only reached a trend in post-hoc tests due to correction for multiple testing. However, of relevance for the reviewer's question is that there is no difference between the 1US and 3US groups, indicating that native Fos expression in TRAP2-tdTom mice does not differ under these conditions. Please note that the similar percentage of ~7% Fos^+ cells in the PL after 1US and 3US CTC corroborates our previous finding in C57Bl/6J mice[1].

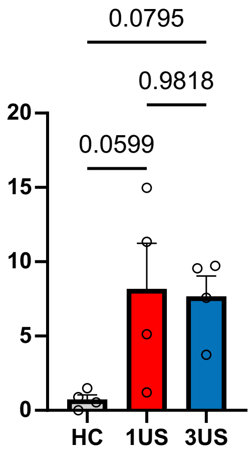

Rebuttal Figure 2. *Fos expression in TRAP2-tdTom mice. One-way ANOVA revealed a significant difference between groups ($F_{2,9}$=4.47; p = 0.04). Post hoc Tukey analyses: HC vs. 1US p = 0.06, HC vs. 3US p = 0.08, 1US vs. 3US p = 0.98)*

Regarding the recombination efficiency, we would like to stress that recombination in TRAP2-tdTom (i.e., TRAP2 x Ai14) mice has been extensively characterized by DeNardo et al., 2019 and that we used the optimal timepoint of 4-OHT injection that was found in their study[2]. It is unfortunately impossible to directly compare tagging and Fos expression induced by training in the same mice. This is due to the difference in temporal expression of Fos protein (peaks 90-120 mins after training) and the Cre recombination-dependent tag (detectable ~12-24 h after training). When comparing Fos (~7%) with tdTomato (1.6-3.9%, depending on the batch), it is evident that the tag reflects a subset of the Fos population in the PL, as also reported by others using TRAP2 x Ai14 reporter mice[2, 3, 7]. However, since both Fos expression and tagging do not differ between 1US and 3US CTC groups, the recombination efficiency is likely the same across experimental conditions.

In our electrophysiological and structural analyses, we did not include home-cage groups, but we included No US (no shock, context only) control groups (Suppl. Fig. 3-5). This condition controls better for changes induced by the handling of mice and exposure to the conditioning context. Also, we show in Fig. 1c that the home-cage group shows less tagged cells compared with the CTC group, making this a less suitable control condition. As we did not find differences in electrophysiological and structural properties between tagged and non-tagged layer 5 pyramidal neurons in the No US group, this further indicates that the differences that we find between tdTom+ and tdTom- neurons are specific for consolidation of aversive memory.

2) Along the same lines, the electrophysiological and synaptic properties of tdTomato-positive neurons should be compared side-by-side across 1US and 3US groups—not just versus randomly selected unlabeled neurons. This would strengthen claims regarding stimulus-specific adaptations.

We understand the remark of the reviewer, but we think that the pairwise within-subject comparisons of tdTom+ and tdTom- PNs are more appropriate based on methodological limitations of the experiments. For electrophysiological analyses, we could record cells from max. 1 mouse per day. To reduce the impact of day-to-day differences in slice conditions, recording variables and placement of the stimulating electrode, we limited our statistical analyses to within-subject pairwise comparisons of tagged and non-tagged neurons. Importantly, selection of tdTom- PNs was not completely random but based on their proximity to tdTom+ PNs in L5 of the PL (as described in the methods section, line 531-532). Due to day-to-day variability, the between group comparisons suggested by the reviewer have unfortunately a higher risk of inflating a type I or type II error. Given that the same cells were used for the structural analyses, we also used within subject comparisons to analyze these data.
To clarify this choice, we added a statement about this in the Methods section:

Line 653-657:
For electrophysiological analyses, we recorded cells from 1 mouse per day. To reduce the impact of small day-to-day differences in slice conditions, recording variables and placement of the stimulating electrode, statistical analyses were limited to within-subject pairwise comparisons of tagged and non-tagged neurons per experimental condition.

We have carefully checked our statements and conclusions, and in agreement with the within-subject comparisons, we only state that 1US tagged neurons differ from 1US non-tagged neurons, or that 3US tagged neurons differ from 3US non-tagged neurons. We do not claim that 1US tagged neurons differ from 3US tagged neurons. For clarity, we adjusted references to the comparisons throughout the manuscript.
Although we think this is the most appropriate way to analyze our datasets, we now acknowledge the limitation of the methodological design to perform direct comparisons between 1US and 3US tagged neurons in a new section of the discussion:

Line 412-419:

Secondly, due to the methodological design of our electrophysiological experiments, we did not directly compare PN populations between conditions (see Methods). Therefore, our conclusions are based only on differences between tagged and non-tagged populations within an experimental condition. Nonetheless, such differences may be required to increase the signal-to-noise ratio in activity of tagged versus non-tagged PNs within the local network, as supported by differences in the reactivation rate of tagged neurons during retrieval of a mild versus strong threat memory.

3) The conclusion that mild stimulation increases structural connectivity is based solely on spine imaging. However, due to the relatively low resolution of optical imaging (especially at 40x with digital zoom), spine counts may be underestimated and spine classification may be inaccurate.

The reviewer raises a valid point. However, we want to mention that the increase in spine density after 1US CTC was observed in independent cohorts of mice with both a 63x objective (Fig. 4) and 40x objective with digital zoom (Fig. 6). Please note that we used the same spine classification parameters as Ortega-de San Luis et al. 2023, and they and others also used a 40x objective for their spine analyses on engram cells[6, 22].
Nonetheless, to determine whether our spine counts and classification could have been influenced by the imaging resolution, we have re-imaged dendritic segments of cells used in Fig. 6 with both a 40x and 100x objective in a counterbalanced order. We acquired images using the Nyquist sampling rate, resulting in a digital zoom of 6.21x and 2.95x, and a resolution of 0.05 and 0.04 µm/pixel for 40x and 100x, respectively. After 3D reconstruction, we found that the overall spine density and the density of stubby, mushroom and long thin spines do not differ between the 40x and 100x objective (Rebuttal figure 3, below), indicating that the use of the 40x objective with digital zoom was sufficient to perform spine classification in our dataset.

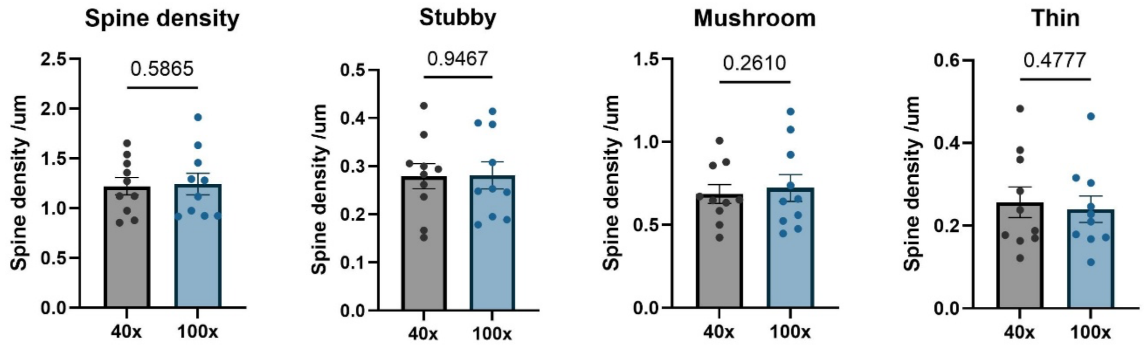

Rebuttal Figure 3, comparison of 40x and 100x objectives. *The overall spine density and density of stubby, mushroom and long thin spines did not differ between images acquired with 40x and 100x objectives. Data were analyzed with a paired t-test. P values are indicated in the graphs.*

More importantly, no evidence is provided to confirm that the newly formed spines are innervated, let alone the identity or origin of presynaptic axons. mEPSC frequency

increases are not necessarily indicative of new functional synapses, especially in light of reported increases in presynaptic release probability.

We agree with the reviewer that we cannot claim with certainty that newly formed spines are innervated. Therefore, we toned-down statements about enhanced connectivity from the abstract, results and discussion.

Other Points

4) The behavioral paradigm used appears to follow standard contextual fear conditioning (CFC). Thus, referring to it as "contextual threat conditioning" may be misleading. Also, the manuscript should consistently use the standard abbreviation "4-OHT" for 4-hydroxytamoxifen.

Fear conditioning and threat conditioning are interchangeably used by the field for the same paradigm[23, 24]. In this study, we used contextual threat conditioning as it more accurately refers to the difference in the external threat (i.e., number of foot-shocks). This is based on the concept proposed by LeDoux and others that fear is a brain construct involving conscious feelings that we cannot measure in rodents[25, 26]. By using threat conditioning instead of fear conditioning, we avoid the interpretation that cellular and synaptic changes are caused by differences in the subjective feeling of fear, but rather through differences in the external threat. For this reason, we have not changed the name of the paradigm. If the reviewer is not convinced by this explanation, we are willing to change it to contextual fear conditioning.

We have replaced '4TM' with '4-OHT' throughout the manuscript.

5) The authors discuss how their findings are supported by prior associative fear engram studies. However, most of those studies used strong conditioning protocols. In particular, Matos et al., 2019 (from one of the corresponding authors) showed that chemogenetic silencing of mPFC engram cells 30 days after 3US reduced freezing, contrary to what is shown in Figure 1f. This discrepancy should be acknowledged and discussed.

This is a misunderstanding of the reviewer. In Matos et al., 2019, we reported that chemogenetic silencing of mPFC engram cells 30 days after strong (3US) training did not reduce freezing levels, in contrast to mild (1US) training. Hence, the present findings in TRAP2-tdTom mice corroborate those findings and therefore form the foundation of our electrophysiological and structural analyses in TRAP2-tdTom mice.

6) It is surprising that a single shock delivered 28 days prior leads to ~80% freezing in most mice (Fig. 1). Are there prior reports showing such robust long-term memory after a single shock? The similarity in freezing between 1US and 3US groups should be contextualized.

We agree that the freezing levels after 1US CTC are relatively high. We noticed that the TRAP2-tdTom mice are generally less active than for instance C57BL/6J mice. Despite these high freezing levels, we are confident that the chemogenetic effect after 1US CTC

is real, because we previously reported the same effect in C57BL/6J mice with controls showing ~62% freezing[1].
To explain the similarity in freezing between 1US and 3US CTC, we added the following text to the results:

Line 113-116:
Remarkably, the 1US group showed high freezing levels which did not differ significantly from the 3US group. We noticed TRAP2-tdTom mice were generally less active than other mouse strains (e.g. C57BL/6J), which likely contributed to the high freezing levels in both groups.

7) Fig. 1b: The staining and image quality are suboptimal and may need improvement for publication.

We kindly want to ask the reviewer whether this comment is based on the figure that was inserted in the manuscript pdf file, or the high-resolution image of Figure 1 that was provided separately with the submission of the manuscript? The image quality of the latter is much better, such that individual cells are visible. All figures are now separately provided as high-resolution versions. If necessary, we can provide images with even higher resolution.

8) Fig. 2: Statistical comparisons should be indicated directly on the graphs, as p-values are not shown in the figure.

Please note that only when statistical comparisons reached significance, we have indicated this in the graphs using asterisks. For clarity, we made the asterisk bigger in Fig. 2j. N.B. this is the only comparison in Fig. 2 that reached statistical significance.

9) Fig. 2a: Pooling recent timepoints (3–7 days) could obscure day-specific variability in excitability, as prior studies in other memory circuits have shown changes across this window.

We thank the reviewer for noticing this error. These recordings were actually performed on day 6-7 after CTC, within the timeframe of recent memory. We have corrected this in the figure. Day-specific variability is therefore limited. Excitability recordings were formed on day 6 or 7 after CTC, because we could only record from one mouse per day. Importantly, we do not find differences in excitability (and synaptic plasticity) at the recent timepoints after 1US and 3US CTC.

10) As mentioned earlier, the current imaging configuration (40x with digital zoom) may lack the resolution necessary for accurate spine classification. Reanalysis using higher magnification optics—or ideally, electron microscopy—would greatly strengthen the conclusions about structural remodeling.

As shown at point 3 (Rebuttal Fig. 3), we re-analysed dendritic fragments with a higher magnification (100x) and compared this with the 40x plus digital zoom. The higher

magnification does not lead to different outcomes for spine density and spine classification. Although electron microscopy has a better resolution, it is less suitable for spine classifications (one would have to use 3D EM) and less feasible for high throughput studies. With EM, we would not have been able to analyze and quantify the large number of dendrites and spines on tagged vs. non-tagged neurons shown in Fig. 6.

11) Introduction, Line 51: The statement that memory retrieval leads to partial reactivation of engram cells should acknowledge that in the cortex, most neurons tagged during learning are not reactivated during retrieval (Roy et al, 2022).

Please note that Roy et al., 2022 studied reactivation of tagged cortical neurons during recent memory retrieval[9]. At that timepoint after training, learning-activated PL neurons are indeed not preferentially reactivated, as shown previously by us and others[1, 6]. Our new data (Fig. 1g-i) confirms that during remote memory retrieval, learning-activated PL neurons are preferentially reactivated.

12) Introduction, Line 64: There is an apparent contradiction between the idea that memory retrieval depends on engram reactivation and the notion that mPFC engrams are disengaged after strong conditioning. If lasting memories rely on engram cells for retrieval, how can they become dispensable after strong training? The logic here needs to be clarified.

We agree that this is an intriguing question. Memories are represented by connected engrams across multiple brain regions and artificial reactivation of engram cells across multiple brain regions is needed to fully resemble freezing levels of natural recall[9]. Since silencing of 3US CTC-tagged PL neurons does not affect memory retrieval, and they are also not reactivated to the same level as 1US CTC-tagged PL neurons, this indicates that these learning-activated cells do not meet the defining criteria of an engram[27]. Therefore, we also do not call them engram cells after a strong threat. In the discussion, we speculate that a strong threat memory relies more on a primordial brain circuit.

Line 298-304:
As the same intervention does not affect expression of a strong threat memory, learning-activated PL neurons do not appear to functionally contribute to the broader, brain-wide engram circuit after a stronger aversive experience. We hypothesize that strong threat memories rely more heavily on evolutionary conserved subcortical brain regions that process emotional stimuli, such as the amygdala, and less on top-down control over these regions by the prefrontal cortex.

13) Introduction, Lines 75–78: The use of vague terms like “adaptations” and “changes” could be replaced with more specific language to improve clarity.

We have changed the text as follows:

Line 76-81:

Our data reveal that threat conditioning-activated PL pyramidal neurons (PNs) develop a time-dependent reduction in postsynaptic strength, mirrored by a decrease in spine size, regardless of threat intensity. In addition, spine density on CTC-activated PNs progressively increased exclusively after mild CTC, whereas the excitability of CTC-activated PNs was reduced compared with non-activated PNs only after strong CTC.

14) Discussion, Lines 428–431: The statement that strong conditioning "disengages" PL engrams should be softened. While chemogenetic silencing data suggest a reduced contribution to remote retrieval, Fos expression still indicates initial engagement, and the persistent physiological changes observed may still influence broader circuit function.

The lack of chemogenetic effect is in line with the reduced reactivation rate of 3US CTC-tagged neurons compared with 1US CTC-tagged neurons (new Fig. 1g-i). This further supports our interpretation that these cells are not engaged in, or at least not functionally contributing to, remote memory retrieval. We have softened this text as follows:

Line 298-301:
As the same intervention does not affect expression of a strong threat memory, learning-activated PL neurons do not appear to functionally contribute to the broader, brain-wide engram circuit after a stronger aversive experience.

15) Discussion, Line 438: The word should be “resilience,” not “resiliance.”

We thank the reviewer for noticing this typo and have corrected it.

16) Discussion, Lines 490–494: The claim that increased spine density after 1US is consistent with interhemispheric PL connectivity reported by Lee et al. (2023) should be reconsidered. That study used 5 shocks (strong conditioning) and reported changes, which contradicts the current claim.

We agree that this extrapolation is inaccurate and have removed that part of the sentence.

17) Several recent studies have shown that Hebbian mechanisms alone cannot explain engram allocation across distributed brain regions. The authors should discuss how their findings relate to this growing body of work.

This is a very relevant point. We agree that Hebbian, or homosynaptic, plasticity does not fully explain engram mechanisms. Therefore, we speculate that our findings related to the reduced eEPSC and spine size may be explained by heterosynaptic plasticity (line 397-399), in line with reports of others[28]. We also changed text in the discussion to stress the importance of non-Hebbian mechanisms:

Line 352-355:
These findings extend previously reported time-dependent increases in spine density on cortical neurons after CTC[6, 29], emphasizing the importance of non-Hebbian synaptic plasticity in engram persistence[20, 28].

Furthermore, we added text to the discussion where we speculate that the enhanced presynaptic release probability after both mild and strong CTC may reflect a form of homeostatic synaptic plasticity:

Line 384-388:
Alternatively, the increase in presynaptic release probability may result from homeostatic synaptic plasticity[16], such that the reduction in postsynaptic strength (through decreased eEPSC amplitude and smaller spines) may be compensated by an increase in the probability of presynaptic vesicle release to maintain synaptic weigth[17].

We think a discussion on non-Hebbian mechanisms that contribute to engram allocation is beyond the scope of our study, as our findings relate to mechanisms of memory consolidation instead of allocation.

18) Additionally, The authors should more thoroughly discuss learning-related changes in excitability and how their results align—or conflict—with earlier studies.

Please note that we discuss earlier studies that investigated the role of excitability in engram allocation and reactivation:

Line 333-336:
Neurons with increased excitability are preferentially recruited into an engram and a transient increase in excitability of hippocampal engram cells after recent memory retrieval determines the efficacy of subsequent memory expression[13, 30].

However, most earlier studies focussed on engram cells in the hippocampus or amygdala. Interestingly, a recently published study examined excitability of layer 5 PNs in the caudal ACC. We now also discuss that study in light of ours:

Line 323-328:
Our data also contrast with the finding that excitability of layer 5 pyramidal engram cells in the caudal anterior cingulate cortex is transiently enhanced within the first 3 days after CTC[31]. The discrepancy may be explained by the difference in brain region or timepoint (d1-3 in their study vs. d6-7 in our study) at which cells were recorded. Transient changes induced by CTC may no longer be detectable several days after learning.

References

1. Matos MR*, et al.* Memory strength gates the involvement of a CREB-dependent cortical fear engram in remote memory. *Nature communications* **10**, 2315 (2019).

2. DeNardo LA*, et al.* Temporal evolution of cortical ensembles promoting remote memory retrieval. *Nat Neurosci* **22**, 460-469 (2019).

3. Golbabaei A, Josselyn SA, Frankland PW. PV-dependent reorganization of prelimbic cortex sub-engrams during systems consolidation. *Neuron*, (2025).

4. Ramaswamy S, Markram H. Anatomy and physiology of the thick-tufted layer 5 pyramidal neuron. *Front Cell Neurosci* **9**, 233 (2015).

5. Anastasiades PG, Carter AG. Circuit organization of the rodent medial prefrontal cortex. *Trends Neurosci* **44**, 550-563 (2021).

6. Kitamura T*, et al.* Engrams and circuits crucial for systems consolidation of a memory. *Science* **356**, 73-78 (2017).

7. Lee JH, Kim WB, Park EH, Cho JH. Neocortical synaptic engrams for remote contextual memories. *Nat Neurosci* **26**, 259-273 (2023).

8. Josselyn SA, Kohler S, Frankland PW. Finding the engram. *Nat Rev Neurosci* **16**, 521-534 (2015).

9. Roy DS*, et al.* Brain-wide mapping reveals that engrams for a single memory are distributed across multiple brain regions. *Nature communications* **13**, 1799 (2022).

10. Alejandre-García T, Kim S, Pérez-Ortega J, Yuste R. Intrinsic excitability mechanisms of neuronal ensemble formation. *eLife* **11**, (2022).

11. Peters KZ*, et al.* Prelimbic cortical excitatory overdrive and inhibitory underdrive accompany environmental suppression of food seeking. *Neuropsychopharmacology*, (2025).

12. Hijazi S*, et al.* Early restoration of parvalbumin interneuron activity prevents memory loss and network hyperexcitability in a mouse model of Alzheimer's disease. *Mol Psychiatry*, (2019).

13. Yiu AP*, et al.* Neurons are recruited to a memory trace based on relative neuronal excitability immediately before training. *Neuron* **83**, 722-735 (2014).

14. Gouty-Colomer LA*, et al.* Arc expression identifies the lateral amygdala fear memory trace. *Mol Psychiatry* **21**, 364-375 (2016).

15. Saviane C, Savtchenko LP, Raffaelli G, Voronin LL, Cherubini E. Frequency-dependent shift from paired-pulse facilitation to paired-pulse depression at unitary CA3-CA3 synapses in the rat hippocampus. *J Physiol* **544**, 469-476 (2002).

16. Zhao C, Dreosti E, Lagnado L. Homeostatic synaptic plasticity through changes in presynaptic calcium influx. *J Neurosci* **31**, 7492-7496 (2011).

17. Delvendahl I, Kita K, Müller M. Rapid and sustained homeostatic control of presynaptic exocytosis at a central synapse. *Proc Natl Acad Sci U S A* **116**, 23783-23789 (2019).

18. Schacter DL. *Forgotten ideas, neglected pioneers : Richard Semon and the story of memory*. Psycholoby Press (2001).

19. Ryan TJ, Roy DS, Pignatelli M, Arons A, Tonegawa S. Memory. Engram cells retain memory under retrograde amnesia. *Science* **348**, 1007-1013 (2015).

20. Uytiepo M*, et al.* Synaptic architecture of a memory engram in the mouse hippocampus. *Science* **387**, eado8316 (2025).

21. Choi J-H*, et al.* Interregional synaptic maps among engram cells underlie memory formation. *Science* **360**, 430-435 (2018).

22. Ortega-de San Luis C, Pezzoli M, Urrieta E, Ryan TJ. Engram cell connectivity as a mechanism for information encoding and memory function. *Curr Biol* **33**, 5368-5380.e5365 (2023).

23. Lesuis SL*, et al.* Stress disrupts engram ensembles in lateral amygdala to generalize threat memory in mice. *Cell* **188**, 121-140.e120 (2025).

24. Casanova JP, Pouget C, Treiber N, Agarwal I, Brimble MA, Vetere G. Threat-dependent scaling of prelimbic dynamics to enhance fear representation. *Neuron* **112**, 2304-2314.e2306 (2024).

25. LeDoux J, Daw ND. Surviving threats: neural circuit and computational implications of a new taxonomy of defensive behaviour. *Nat Rev Neurosci* **19**, 269-282 (2018).

26. LeDoux JE. *Anxious : using the brain to understand and treat fear and anxiety*. Viking (2015).

27. Josselyn SA, Köhler S, Frankland PW. Finding the engram. *Nat Rev Neurosci* **16**, 521-534 (2015).

28. Bhasin BJ, Raymond JL, Goldman MS. Synaptic weight dynamics underlying memory consolidation: Implications for learning rules, circuit organization, and circuit function. *Proc Natl Acad Sci U S A* **121**, e2406010121 (2024).

29. Vetere G*, et al.* Spine growth in the anterior cingulate cortex is necessary for the consolidation of contextual fear memory. *Proc Natl Acad Sci U S A* **108**, 8456-8460 (2011).

30. Pignatelli M*, et al.* Engram Cell Excitability State Determines the Efficacy of Memory Retrieval. *Neuron* **101**, 274-284 e275 (2019).

31. Hadzibegovic S*, et al.* Early intrinsic excitability plasticity of neocortical engram neurons defines memory formation and precision. *Nature communications* **17**, 291 (2025).
